# Genetic Mapping of Prince Rupprecht’s Larch (*Larix principis-rupprechtii* Mayr) by Specific-Locus Amplified Fragment Sequencing

**DOI:** 10.3390/genes10080583

**Published:** 2019-07-31

**Authors:** Mingliang Dong, Qingwei He, Jian Zhao, Yan Zhang, Deshui Yuan, Jinfeng Zhang

**Affiliations:** 1 Beijing Advanced Innovation Center for Tree Breeding by Molecular Design, National Engineering Laboratory for Tree Breeding, Key Laboratory of Genetics and Breeding in Forest Trees and Ornamental Plants of Ministry of Education, Key Laboratory of Forest Trees and Ornamental Plants Biological Engineering of State Forestry Administration, College of Biological Sciences and Biotechnology, Beijing Forestry University, Beijing 100083, China; 2 National Key Seed Base of Larch, Weichang, Chengde 068450, China

**Keywords:** genetic map, SLAF-seq, SNP, larch, *Larix principis-rupprechtii*

## Abstract

A high-density genetic linkage map is essential for plant genetics and genomics research. However, due to the deficiency of genomic data and high-quality molecular markers, no genetic map has been published for Prince Rupprecht’s larch (*Larix principis-rupprechtii* Mayr), a conifer species with high ecological and commercial value in northern China. In this study, 145 F1 progeny individuals from an intraspecific cross between two elite clones of *L. principis-rupprechtii* and their parents were employed to construct the first genetic map in this important tree species using specific-locus amplified fragment sequencing (SLAF-seq). After preprocessing, the procedure yielded 300.20 Gb of raw data containing 1501.22 M pair-end reads. A total of 324,352 SNP markers were detected and 122,785 of them were polymorphic, with a polymorphism rate of 37.86%. Ultimately, 6099 SNPs were organized into a genetic map containing 12 linkage groups, consistent with the haploid chromosome number of larch and most other species in the Pinaceae family. The linkage map spanned 2415.58 cM and covered 99.6% of the *L. principis-rupprechtii* genome with an average of 0.4 cM between adjacent markers. To the best of our knowledge, this map is the first reference map for *L. principis-rupprechtii*, as well as the densest one obtained in larch species thus far. The genome-wide SNPs and the high-resolution genetic map will provide a foundation for future quantitative trait loci mapping, map-based cloning, marker-assisted selection, comparative genomics, and genome sequence assembly for larch trees.

## 1. Introduction

Prince Rupprecht’s larch (*Larix principis-rupprechtii* Mayr, 2n = 24), as a member of the genus *Larix*, plays a crucial role in reforestation programs and commercial activities in North China due to its superior biological characteristics, including rapid growth at early ages, wide ecological adaptability, and desirable wood properties [1,2]. The economic importance and ecological benefits of this species have prompted breeders to carry out genetic improvement measures based on recurrent selection schemes, with the ultimate goal of developing new varieties characterized by fast growth, high timber quality, and strong disease resistance [3]. However, this is a complicated process and difficult to achieve in a short time using traditional breeding methods (e.g., cross breeding, recurrent selection), mainly because of the long lifecycle and highly heterozygous nature of *L. principis-rupprechtii*, like other conifers [4]. The vast majority of economically important traits in forest trees are quantitatively inherited, controlled by a number of minor-effect genes and influenced by environmental factors [5], which further hinder the breeding of *L. principis-rupprechtii*. By comparison, marker-assisted selection (MAS) is an ideal approach to improve the breeding efficiency and shorten the selection times of plants for its independence from the external environment and direct selection of genotypes with target traits in an early stage using trait-linked markers [6,7]. A prerequisite for successfully implementing MAS is the use of genomic tools, particularly genetic linkage maps with high quality.

A linkage map indicates the relative order and genetic distances between markers along chromosomes as inferred by segregation information in a mapping population derived from a specific cross [8,9]. It is a necessary tool for quantitative trait locus (QTL) mapping, identification of candidate genes, and comparative genomics, and facilitates genome assembly and MAS [10,11]. As a group of perennial woody plants, long-term outcrossing of larch species causes high levels of heterozygosity in their genetic composition, making it extremely difficult to build a traditional mapping population as widely used in crops [12]. Meanwhile, the gigantic and complex conifer genome, characterized by great regions of repeatable elements [13,14], offers challenges for polymorphism genotyping, and draft genome assembly. These attributes have contributed to the difficulty in linkage map construction of larch species. To the best of our knowledge, there are only two reports on the construction of genetic maps in the genus *Larix*. Arcade et al. [15] constructed single-tree genetic linkage maps of *L. decidua* (female parent) and *L. kaempferi* (male parent) based on 112 F1 individuals from an interspecific cross using 114 amplified fragment length polymorphism (AFLP), 149 random amplified polymorphic DNA (RAPD), and 3 inter simple sequence repeat (ISSR) markers, with average distances of 13.8 cM in the female map and 14.0 cM in the male map. Subsequently, parent-specific linkage maps using 145 F1 plants derived from the cross of *L. kaempferi* × *L. gmelini* were developed [16], which contained 210 RAPD markers with an average marker spacing of 11.9 and 6.1 cM for the maternal and male parent, respectively. The numbers and types of polymorphic markers used for linkage mapping determine the utility of a genetic map to some extent [17]. Thus, the poor-quality maps constructed by the two previous studies are less useful for fine mapping of desired traits for MAS to accelerate breeding. In addition, these types of markers are incompatible with future genome anchoring owing to the lack of sequence information. As powerful co-dominant markers, simple sequence repeats (SSR) markers have been applied in multiple genetic studies of larch, such as of parentage [2,18], genetic diversity [19], and genetic relationships among clones [3]. Although some cross-species and species-specific SSR markers for *L. principis-rupprechtii* have been developed [3,20], the small number of markers available, along with the low polymorphic marker ratio [21] and the huge genome of *Larix* (approximately 13.2 Gb) [22], hampers generation of a high-resolution SSR-based genetic map for this conifer species.

To create high-density genetic maps, researchers have transferred their attention from traditional molecular markers to single nucleotide polymorphism (SNP) markers due to their high abundance and uniform distribution across the genome [23,24]. By combining next-generation sequencing (NGS) technology with reduced representation libraries (RRLs), specific-locus amplified fragment sequencing (SLAF-seq) enables large-scale SNP discovery and genotyping of mapping populations to generate high-density linkage maps in a low-cost but efficient manner [25,26]. In addition, SLAF-seq does not rely on a reference genome sequence, which is a particularly attractive feature for species whose genomes have not yet been sequenced [25]. This technology has been successfully applied to generate high-density genetic maps in several tree species, such as tea plant [27], *Prunus mume* [28], mango [26], *Salix matsudana* [29], sweet osmanthus [30], and *Ginkgo biloba* [31], but not yet for *L. principis-rupprechtii*. Therefore, construction of such a SNP-based genetic linkage map using SLAF-seq is needed.

In the current study, a full-sibling mapping population containing 145 F1 individuals was established from intraspecific hybridization between two elite clones of *L. principis-rupprechtii*. SLAF-seq was applied for genome-wide SNP identification and genotyping in this segregation population, and the generated genotype data were analyzed to construct the first high-density genetic map of *L. principis-rupprechtii*. The large-scale SNP markers reported here will facilitate further genetic studies and breeding of *L. principis-rupprechtii*, and the high-density genetic map will provide a powerful genomic tool for QTL fine mapping of complex and important traits for MAS to promote genetic improvement of this valuable conifer species.

## 2. Materials and Methods

### 2.1. Plant Material and DNA Isolation

To develop an F1 mapping population of *L. principis-rupprechtii*, an intraspecific cross was made between the elite clones No. 9 and No. 100, using No. 9 as the female parent. From the perspective of larch breeding, a cross between two excellent genotypes would enable selection of superior progenies from the resulting segregation population. The hybridization was performed at the National Key Seed Base of Larch (41°56′ N, 117°45′ E), Weichang County, Hebei Province, China, in April 2017. Mature cones were harvested about 4 months later and transported to the greenhouse of Beijing Forestry University (39°09′ N, 116°03′ E, Beijing). These cones were kept dry at room temperature until opened. After being manually extracted from the cones, the seeds were allowed to germinate at 25 °C for 1 week, and then those with radicles at least 2 mm long were planted in potting soil (peat:perlite:vermiculite = 2:1:1) under continuous lighting conditions. After 2 months of growth, robust and healthy seedlings were transferred to individual containers. Finally, 145 F1 progeny plants were selected for the construction of genetic linkage map.

Young needles from the parents and progenies were sampled after 4 months of growth and stored at −80 °C until use. Genomic DNA was isolated using a modified CTAB method as described by Doyle and Doyle [32]. The extracted DNA was resolved on 0.8% agarose gels and its purity and concentration were analyzed using a NanoDrop 2000 spectrophotometer.

### 2.2. SLAF Library Construction and High-Throughput Sequencing

The SLAF-seq strategy [25] with some modifications was used in this study. The restriction enzyme *Hae*III (New England BioLabs, NEB, Ipswich, MA, USA) was used to digest the genomic DNA of the 145 F1 progenies and 2 parents. Subsequently, an A-overhang was added to the digested fragments by Klenow Fragment (3′ → 5′ exo-) (NEB) and dATP. Thereafter, T4 DNA ligase (NEB) was used to ligate the polyacrylamide gel electrophoresis (PAGE)-purified duplex tag-labeled sequencing adapters (Life Technologies, Carlsbad, CA, USA) to the A-tailed fragments. Polymerase chain reaction (PCR) amplification was carried out using a reaction mixture containing dNTP, PCR primers (Forward primer: 5′-AATGATACGGCGACCACCGA-3′, reverse primer: 5′-CAAGCAGAAGACGGCATACG-3′), diluted restriction-ligation DNA samples and Q5^®^ High-Fidelity DNA Polymerase. After being purified using Agencourt AMPure XP beads (Beckman Coulter, High Wycombe, UK) and pooled, the amplification products were resolved on a 2% agarose gel. Fragments of 414–444 bp, including the index and adaptor were excised and cleaned using a QIAquick Gel Extraction Kit (Qiagen, Hilden, Germany), and the gel-purified products were diluted and subjected to paired-end sequencing (125 bp each end) on an Illumina HiSeq 2500 system (Illumina, Inc., San Diego, CA, USA) at the Biomarker Biotechnology Corporation (Beijing, China).

The SLAF-seq strategy [25] with some modifications was used in this study. The restriction enzyme *Hae*III (New England BioLabs, NEB, Ipswich, MA, USA) was used to digest the genomic DNA of the 145 F1 progenies and 2 parents. Subsequently, an A-overhang was added to the digested fragments by Klenow Fragment (3′ → 5′ exo-) (NEB) and dATP. Thereafter, T4 DNA ligase (NEB) was used to ligate the polyacrylamide gel electrophoresis (PAGE)-purified duplex tag-labeled sequencing adapters (Life Technologies, Carlsbad, CA, USA) to the A-tailed fragments. Polymerase chain reaction (PCR) amplification was carried out using a reaction mixture containing dNTP, PCR primers (Forward primer: 5′-AATGATACGGCGACCACCGA-3′, reverse primer: 5′-CAAGCAGAAGACGGCATACG-3′), diluted restriction-ligation DNA samples and Q5^®^ High-Fidelity DNA Polymerase. After being purified using Agencourt AMPure XP beads (Beckman Coulter, High Wycombe, UK) and pooled, the amplification products were resolved on a 2% agarose gel. Fragments of 414–444 bp, including the index and adaptor were excised and cleaned using a QIAquick Gel Extraction Kit (Qiagen, Hilden, Germany), and the gel-purified products were diluted and subjected to paired-end sequencing (125 bp each end) on an Illumina HiSeq 2500 system (Illumina, Inc., San Diego, CA, USA) at the Biomarker Biotechnology Corporation (Beijing, China).

### 2.3. Analyses of SLAF-Seq Data and Identification of SNP Loci

Data for SLAF-seq were analyzed as detailed in Sun et al. [25]. In brief, low-quality paired-end reads (quality score < 20) were discarded and the remaining reads were demultiplexed to 145 progeny individuals based on duplex barcode sequences. Next, the barcodes and the terminal 5 bp position were removed from the high-quality reads, and 100 bp paired-end clean reads were clustered using a sequence similarity of >90%. Clustered sequences were considered one SLAF locus [28]. According to the number of tags in one locus, the SLAFs could be divided into three types: non-polymorphic SLAF, polymorphic SLAF, and repetitive SLAF. Because *L. principis-rupprechtii* is a diploid species, a SLAF can harbor up to four genotype tags. Hence, non-polymorphic SLAFs embracing only one tag, and repetitive SLAFs containing more than four tags, were discarded. Only SLAFs with two to four tags were considered potential polymorphic markers. SNPs were detected based on the polymorphism information of SLAF tags using GATK [33] and SAMtools [34] software. To ensure the accuracy of SNP calling, only SNPs detected by both methods and parental sequence depths of greater than four-fold were considered reliable. These SNP markers were grouped into four segregation patterns (aa × bb, hk × hk, lm × ll, and nn × np). As the F1 mapping population was derived from a cross between two heterozygote parents, the SNPs with segregation patterns of aa × bb were filtered out. Before carrying out map construction, SNPs were screened using the following criteria. First, SNPs with sequencing depths <10-fold in the parents were excluded. Second, a SNP should be called in at least 75% of individuals. Third, any marker with significant segregation distortion (chi-square tests, *p* < 0.01) was eliminated from the SNP dataset.

### 2.4. Construction of a Linkage Map

To generate a high-density genetic map of *L. principis-rupprechtii*, the HighMap strategy described by Liu et al. [35] was applied. Briefly, high-quality SNPs were assigned to linkage groups (LGs) based on pairwise modified logarithm of odds (MLOD) scores >6. Subsequently, spatial sampling, enhanced Gibbs sampling, and simulated annealing algorithms [36,37] were used to order SNP markers and estimate map distances. Subsequently, the incorrect genotypes were identified and eliminated from the data using the k-nearest neighbor algorithm [38,39]. An iterative process of SNP ordering and error genotype correction was conducted to arrange the markers in the correct order. After several cycles, accurate linkage maps were obtained. The Kosambi mapping function [40] was used in the process. The expected genome length was estimated according to method 4 described by Chakravarti et al. [41], where the observed length of each LG is multiplied by (m + 1)/(m − 1), with m being the number of markers in that LG. The genome coverage is the ratio of the total observed and expected genome lengths [42]. Finally, haplotype maps and heat maps were generated to evaluate the quality of the map.

## 3. Results

### 3.1. Analyses of SLAF-Seq Data

**Figure 1:**
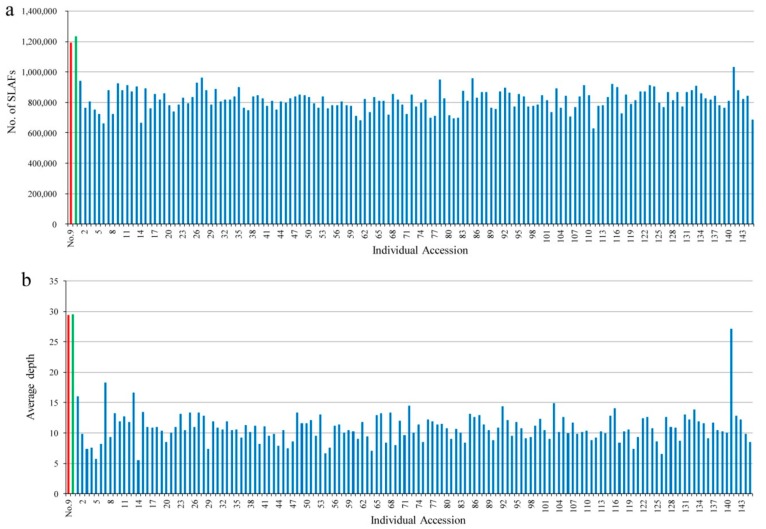
Number and depth of SLAFs for each F1 individual and its two parents. Horizontal axes in (**a**,**b**) indicate individual accessions represented by different colors, red: parent no. 9, green: parent no. 100, blue: F1 progeny individuals from 1 to 145; the vertical axis indicates the number and average depth of SLAFs in (**a**) and (**b**), respectively.

After preprocessing, 300.20 Gb of raw data containing 1501.22 M pair-end reads were obtained. For these reads, 94.22% of sequencing bases were high-quality, with quality scores of ≥30 (Q30, indicating a 0.1% error rate), and the average guanine-cytosine (GC) content was 40.78%. Given the importance of parents in mining segregating markers, the two parents were subjected to sequencing at a higher level than their progeny. Hence, the number of reads in the maternal and paternal parents was 38,277,616 and 39,316,097, respectively, while that for the 145 progeny individuals ranged from 4,097,675 to 30,302,687 with an average of 9,818,123 (Table S1). Sequence alignment and clustering generated a total of 6,323,943 SLAFs, of which 1,193,926 and 1,234,200 were identified in the female and male parents; their corresponding sequencing depths were 29.45- and 29.53-fold, respectively. In the F1 mapping population, the number of SLAFs for each progeny ranged from 628,807 to 1,031,235 with an average of 816,026, and the mean SLAF sequencing depth was 10.88-fold, with a range of 5.55- to 27.16-fold (Figure 1). The raw sequence data have been submitted to the National Center for Biotechnology Information Short Read Archive with project accession of PRJNA541022.

### 3.2. Identification of SNP Markers

**Figure 2:**
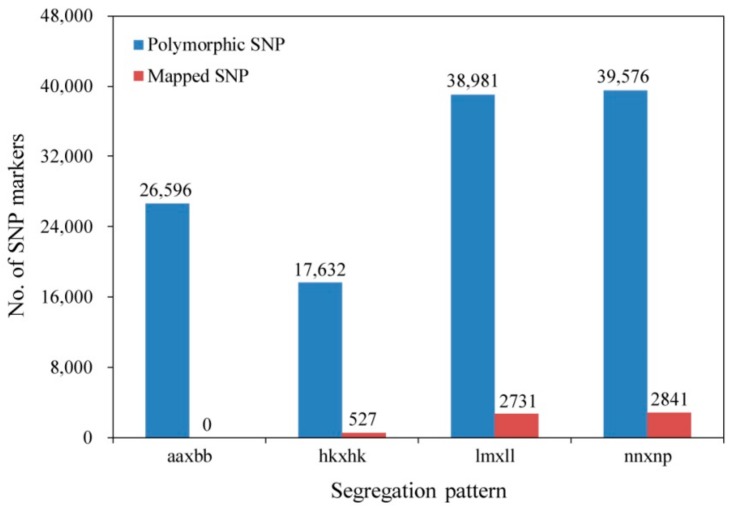
Number of polymorphic SNPs and final mapped SNPs in four segregation patterns.

The sequence with the highest copy number for each SLAF was selected as the reference sequence and was used to develop SNP markers. There were 764,485 and 850,849 SNPs in female and male parents, respectively, and the average number of SNPs for each progeny individual was 492,567 (Table S2). After filtering out SNPs with a sequencing depth in the two parents of less than four-fold, 324,352 markers were detected, 122,785 of which were polymorphic, a polymorphism rate of 37.86%. The polymorphic SNPs were successfully genotyped into four segregated patterns (Figure 2). Because the aa × bb segregation type was not suitable for the F1 population, 96,189 SNPs from the remaining three patterns (hk × hk, lm × ll, and nn × np) were used for further analyses. After filtering out low-quality SNPs with parental sequence depths of <10-fold, integrities of <75%, and significant segregation distortion (*p* < 0.01), 6931 markers were defined as valid and used to construct the genetic map.

### 3.3. Construction of the Linkage Map

**Figure 3:**
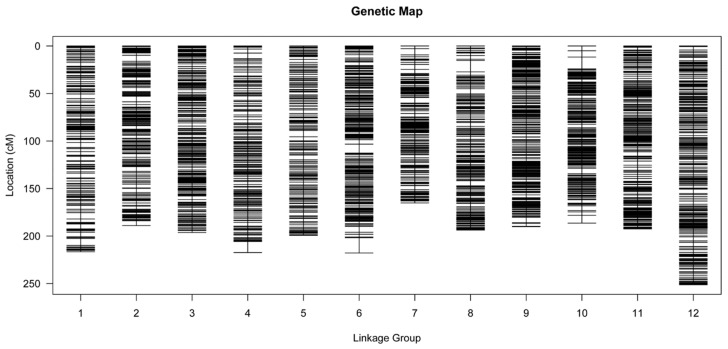
Distribution of SNP markers among the 12 linkage groups of *L. principis-rupprechtii*. Black line, SNP marker. The linkage group number and genetic distance (cM) are shown on the *x*- and *y*-axes, respectively.

Of the 6931 SNPs available for genetic map construction, 6099 (88.00%) were mapped onto 12 LGs based on the MLOD values between markers and the linkage analyses (Figure 2, Tables S3 and S4). The average sequencing depth of the anchored markers was 66.81-fold in the female parent, 64.86-fold in the male parent, and 17.73-fold in each F1 progeny (Table 1). The average integrity of these markers was 99.98%, indicating that the map was high quality. Finally, an integrated linkage map of 2415.58 cM in total length and 0.40 cM in average length was created (Table 2, Figure 3). Detailed information on this map is presented in Table S5. The map contained 12 LGs that were equal to the haploid chromosome number of *L. principis-rupprechtii*, but differed in the length and the distribution of markers. LG12 was the longest (251.28 cM) and had the most markers (731 SNPs), whereas LG7 was the shortest (165.15 cM) and had the fewest markers (301 SNPs). LG7 also had the lowest average inter-marker distance of 0.55 cM, whereas LG6 was the most saturated with an average marker density of 0.33 cM. The degree of linkage between adjacent loci in each LG was reflected by the ‘Gap < 5 cM’ value, which ranged from 98.57% (LG10) to 100% (LG3, LG11, and LG12), with an average of 99.51%. The largest gap with no marker coverage was located in LG6 and spanned a genetic distance of 16.05 cM (Table 2). The total expected genome length was estimated to be 2425.38 cM, ie the integrated map constructed in this study covered 99.6% of the genome. Sex-specific maps were also constructed in this study. The female map consisted of 3368 markers with a mean of 281 markers per LG, and spanned 2275.33 cM with an average marker interval of 0.68 cM, whereas the male map consisted of 3258 markers with a mean of 272 markers per LG, and spanned 2393.62 cM with an average marker interval of 0.74 cM. The basic features of the LGs in the two parental maps are listed in Table 3 and Table S5.

### 3.4. Evaluation of the Genetic Map

The quality of the *L. principis-rupprechtii* genetic map was evaluated by generating haplotype and heat maps. Haplotype maps, which can reveal double crossovers caused by genotyping errors, were created for the parental controls and 145 F1 progenies using 6099 SNP markers (Figure S1). In the haplotype maps, the recombination events for each individual were intuitively shown and most recombination blocks were distinctly defined. Moreover, a low percentage of missing data (<0.01%) was found for each LG of the integrated linkage map. Most LGs were uniformly distributed, suggesting that the map was high quality. After calculating the pairwise recombination scores for the 6099 mapped SNPs, heat maps were generated to visualize the recombination relationship between markers and identify any ordering errors present in each LG. The rate of recombination between two markers increased with increasing genetic distance (Figure S2), indicating that the SNP markers in most LGs were ordered correctly. Consequently, the genetic map of *L. principis-rupprechtii* was of high quality.

## 4. Discussion

A high-density genetic map was constructed based on an F1 mapping population derived from an intraspecific cross of two elite clones deployed in a clonal seed orchard of *L. principis-rupprechtii*. The resulting F1 segregating population was not only the basis for the construction of a dense linkage map but also may promote the generation of excellent progeny individuals with desirable characteristics and provide information regarding inheritance of important growth and wood quality traits. Different from previously published genetic maps in other larch species, the molecular markers used here were SNP markers detected by SLAF-seq, which enables identification and genotyping of SNPs in species with or without reference genomes [25]. Compared to conventional methods for developing markers, SLAF-seq yields higher-density and more uniform and efficacious markers at lower cost, due to its use of a predesigned scheme, reduced representation strategy, NGS-based deep sequencing, and strict filtering criteria [43,44]. To date, SLAF-seq technology has been applied in studies of several tree species. For instance, Ma et al. [27] applied SLAF-seq to identify a total of 6042 valid SNPs, and mapped them into the previous SSR-based framework map of tea plant. Su et al. [45] conducted large-scale detection of SNPs in Chinese fir using SLAF-seq, and used a set of 48,406 informative SNPs to clarify the genetic relationships among 18 parent clones. Liu et al. [31] employed SLAF-seq to detect 538,031 high-quality SLAFs, and used 12,263 of them to construct a highly dense genetic map of *G. biloba*. Here, SLAF-seq was used to identify reliable SNP markers in *L. principis-rupprechtii*. In total, 1501.22 M paired-end reads and 6,323,943 SLAFs were generated. From these SLAFs, 324,352 SNP markers were detected, 122,785 of which were polymorphic. The polymorphism rate of markers (37.86%) was comparable to that reported for other tree species, such as *P. mume* (40.35%) [28], *S. matsudana* (35.89%) [29], and *Jatropha curcas* (38.15%) [46], but higher than for tree peony (27.5%) [47], tea plant (30.4%) [27], and European aspen (30%) [48]. Thus, there was a moderate level of polymorphism between the two parental clones, in agreement with several previous studies of genetic diversity in *L. principis-rupprechtii* [3,49]. The considerable genetic differences exhibited by the two parents suggest that they were suitable for the construction of a segregation population to carry out genetic linkage mapping.

Despite the advantages of NGS strategies for genome-wide SNP discovery, NGS data contain a large amount of missing data and errors [50], particularly that of complex conifer genomes with high-copy number repeat elements [23]. Therefore, to screen a sufficient number of robust SNPs to construct a high-quality genetic map of *L. principis-rupprechtii*, we adopted a series of strict criteria during SNP marker development and genotyping, including filtering out SNPs with a sequencing depth of <10-fold, marker integrity of <75%, and deviation from the expected Mendelian ratio. It should be noted that although segregation distortion markers could increase the genome coverage and might improve QTL mapping [51,52], to reduce computational complexity and ensure construction of an accurate map, these markers were excluded from our analyses, as in prior works [11,53]. After filtering and linkage analyses, 6099 of the 122,785 polymorphic SNPs were mapped onto the integrated linkage map with an average sequencing depth of 65.84- and 17.73-fold for the parents and progeny, respectively, and a mean integrity of >99% for all samples. These parameters indicate the reliability and accuracy of the SNP markers for genetic mapping, and the genome-wide SNPs reported here will facilitate germplasm identification, genetic diversity evaluation, and comparative genomic studies on *Larix* species. Thus, SLAF-seq is an efficient method for large-scale SNP identification, even in conifer species with a huge and complex genome, such as *L. principis-rupprechtii*.

Genetic maps provide a basic platform for QTL analyses of the important economic traits targeted by genetic improvement programs. In view of the great economic and ecological benefits of larch trees, researchers have made significant efforts to construct linkage maps in the past decade. However, due to the complexity of the genome and the paucity of available molecular markers, as far as we know, only two studies in which larch linkage maps were constructed have been published [15,16]. The principal drawback of these maps was their inclusion of only several hundred markers, which resulted in low resolution and thus hindered their utilization for QTL mapping. Reductions in the cost of high-throughput sequencing and increases in data processing capacity have facilitated the construction of high-density genetic maps. In this study, SLAF-seq was used to generate an integrated linkage map of *L. principis-rupprechtii*. This map contained 12 LGs, consistent with the haploid chromosome number of larch and most other species in the family Pinaceae [22]. In addition, the map consisted of 6099 SNP markers and spanned 2415.58 cM, with an average distance of 0.40 cM between adjacent markers. Compared to previous linkage maps (see Introduction) [15,16], a larger number of mapped markers with known sequence information as well as a higher map resolution with a smaller average marker interval was produced in this study. The total length of genetic linkage maps of larch species ranges from 262.0 cM in *L. gmelini* [16] to 2997.0 cM in *L. kaempferi* [15]. The variation in map length may be due to several factors, including differences in mapping population size, marker number, and type; genotyping accuracy; and the software and algorithms used for linkage analyses. In terms of marker density, our linkage map was not only the densest among larch species, but was also denser than the genetic maps of other conifer species to date. For example, the marker densities in recently reported genetic maps of conifers are 0.58–0.62 cM for *Pinus taeda* [54,55], 2.80 cM for *Picea abies* [14], 0.93 cM for *P. pinaster* [56], 0.6 cM in a consensus map of *P. taeda* and *P. elliottii* [57], and 0.47–1.1 cM for *Cryptomeria japonica* [58,59]. Nevertheless, the map density is lower than that reported by Sakaguchi et al. [23], likely due to use by the latter of an imputation procedure to decrease the mean inter-marker distance in *Callitris glaucophylla* from 1.01 cM to 0.24 cM. The mapped SNP markers were almost evenly distributed on the map, except for a few positions on the LGs (e.g., a maximum gap of 16.05 cM at the end of LG6). The presence of large gaps is a common phenomenon in linkage mapping using reduced-representation genome sequencing and may be caused by high levels of recombination, a lack of marker polymorphism, or failure to detect markers in the corresponding genomic regions [47,60]. Given the potential negative impact of these large gaps on genomic studies, measures should be implemented to map more markers in these regions to improve the resolution of the linkage map. These measures include expansion of the current mapping population size, use of other restriction enzymes in library preparation, and construction of consensus genetic maps based on multiple populations. In our opinion, the last measure has several advantages. First, mapping of molecular markers using multiple populations can provide a high level of genome coverage and reduce the number of large gaps, as multiple parents with different genetic backgrounds are unlikely to be monomorphic in the same genomic regions. Second, use of multiple mapping populations increases the overall population size and thus improves the likelihood of capturing a greater number of recombination events [11,61]. The present map covered 99.6% of the estimated genome length. The ratio was comparable to or slightly higher than the values of 94–99% in *P. taeda* [55,62], 96.5% in *P. abies* [14], and 99.0% in *C. japonica* [58]. Therefore, this map should be saturated enough to allow for comprehensive QTL detection. The collinearity between physical and genetic distances of all mapped markers in each LG is important for evaluating the quality of the genetic map [28]. However, because the reference genome of larch has not been released, collinearity analyses could not be performed. Furthermore, the quality of the map can be validated by comparing the order of the common markers between different genetic maps [9]. However, this was not possible due to the lack of shared markers with previous larch maps. Finally, our map was evaluated visually by means of haplotype maps and heat maps. The results showed that the majority of LGs were distributed uniformly, and the SNP markers in most LGs were well-ordered, indicating the linkage map to be of high quality. Thus, SLAF-seq is suitable for constructing high-density linkage maps for *L. principis-rupprechtii*. Such maps will facilitate QTL mapping of economically important traits and molecular breeding of this valuable conifer species.

Genotyping with polymorphic molecular markers is a key step in the construction of genetic maps. Recently, various genotyping methods—such as whole-genome sequencing (WGS), reduced representation genome sequencing (RRGS), RNA sequencing, exome capture, and SNP array—have been widely used in linkage mapping in species with or without available genetic information [30,55,63,64,65]. Although the cost of sequencing is falling, population-scale WGS is still not affordable for conifers due to their huge genome sizes and complexity [23]. RRGS, which reduces the genome complexity by only sequencing DNA fragments after digestion with different restriction enzymes, is a cost-effective alternative for SNP detection and genotyping. As two representative methods of RRGS, SLAF-seq enables greater specificity, consistency, and accuracy in marker development when compared to genotyping-by-sequencing (GBS) [25,66]. RNA sequencing and exome capture focus on the expressed regions of the genome. The gene-based markers mined by these two approaches could themselves be causative markers for one trait, which is particularly important in QTL mapping studies for species with fragmented genome sequences [14]. Additionally, gene-based linkage maps can be used in synteny comparisons between related species to provide insights into genome evolution [64]. However, because the expressed regions only account for a small part of the genome, the gene-based map may have relatively low genome coverage. Compared with the above sequencing-based techniques, SNP arrays can avoid repeated marker screening when genotyping new populations [65], but it also faces problems such as low rates of SNP conversion and prone to ascertainment bias [67,68]. Therefore, appropriate genotyping methods should be selected based on the genetic background of the species, research foundation and purposes, technique characters, and so on. The new mapping strategy and data analysis methods should greatly promote the linkage mapping for highly heterozygous outcrossing species [69].

In summary, we developed a novel set of high-quality SNP markers for *L. principis-rupprechtii* using SLAF-seq. Overall, 122,785 polymorphic SNPs were identified by genotyping of 2 elite parental clones and their 145 F1 progeny individuals. A subset of 6099 SNPs was used to construct a high-density integrated linkage map of the intraspecific hybrid population described above. Taken together, our findings demonstrate the utility of SLAF-seq for the discovery of large-scale SNP markers and the construction of high-density genetic maps for larch species lacking a reference genome. To the best of our knowledge, the linkage map generated in this study is not only the first reference map for *L. principis-rupprechtii*, but is also the densest map for larch species reported to date. The SNP-based high-density linkage map will be useful for the next phase of genetics and breeding research on larch, such as QTL mapping, map-based cloning, comparative genome analyses, and MAS. In addition, SNP markers in this map were mined at the whole-genome level and the sequence and the locations of the genome-wide markers were available. Thus, our high-resolution genetic map will also facilitate orientation of sequence scaffolds and assembly of the draft genomes of larch species.

## Figures and Tables

**Table 1:** Sequencing depths of the SNPs in the map.

Samples	SNP Numbers	Total Depth	Average Depth
No. 9 (female parent)	6099	407,497	66.81
No. 100 (male parent)	6099	395,553	64.86
Average of 145 F1 progenies	5844	103,617	17.73

**Table 2:** Basic characteristics of the integrated linkage map of *L. principis-rupprechtii*.

LG ID	Marker Number	Total Distance (cM)	Average Distance (cM)	Gap < 5 cM (%) ^a^	Max Gap (cM)
LG1	509	216.51	0.43	99.21	7.26
LG2	408	188.99	0.46	99.26	6.36
LG3	527	196.28	0.37	100.00	3.71
LG4	543	217.39	0.40	99.63	11.59
LG5	530	199.24	0.38	99.81	6.72
LG6	655	217.80	0.33	99.54	16.05
LG7	301	165.15	0.55	99.00	6.54
LG8	425	193.74	0.46	99.29	12.02
LG9	538	190.22	0.35	99.81	5.84
LG10	422	186.42	0.44	98.57	12.47
LG11	510	192.56	0.38	100.00	4.41
LG12	731	251.28	0.34	100.00	4.59
Total	6099	2415.58	0.40	99.51	16.05

^a^ Percentages of gaps in which the distance between the adjacent markers was less than 5 cM.

**Table 3:** Basic features of the maps for female and male *L. principis-rupprechtii*.

LG ID	Marker Number		Total Distance (cM)		Average Distance (cM)		Gap < 5 cM (%) ^a^		Max Gap (cM)	
	Female	Male	Female	Male	Female	Male	Female	Male	Female	Male
LG1	285	281	197.79	232.45	0.70	0.83	96.48	95.00	13.36	16.14
LG2	188	254	180.38	191.83	0.96	0.76	95.72	96.44	20.10	9.05
LG3	300	252	177.57	199.64	0.59	0.80	97.99	96.81	7.42	20.10
LG4	298	288	195.35	226.51	0.66	0.79	97.31	95.82	8.23	16.14
LG5	287	299	186.03	212.45	0.65	0.71	97.55	95.30	10.73	9.05
LG6	337	380	227.04	180.53	0.68	0.48	97.92	98.94	16.05	20.10
LG7	207	110	188.70	123.83	0.92	1.14	97.57	96.33	6.54	19.08
LG8	239	218	155.43	228.57	0.65	1.05	98.74	95.39	28.92	20.10
LG9	306	277	186.26	192.07	0.61	0.70	98.69	98.91	5.85	10.73
LG10	216	244	136.35	186.99	0.63	0.77	98.60	98.35	11.59	12.47
LG11	303	258	177.34	196.50	0.59	0.76	99.01	99.22	11.59	20.10
LG12	402	397	267.09	222.25	0.67	0.56	99.00	98.23	22.21	8.10
Total	3368	3258	2275.33	2393.62	0.68	0.74	97.88	97.06	28.92	20.10

^a^ Percentages of gaps in which the distance between the adjacent markers was less than 5 cM.

## Supplementary Materials

The following are available online at https://www.mdpi.com/2073-4425/10/8/583/s1, Table S1: Summary of the SLAF-seq data. Table S2: Number of SNP markers detected in parents and each progeny individual. Table S3: Sequence information of the SNP markers on the map. Table S4: Segregation data of 6099 SNP loci in 145 individuals. Table S5: SNP markers and their genetic positions in each linkage group. Figure S1: Haplotype maps of the linkage map. Each row represents a marker arranged according to the map order. Each of the two columns represents an individual; two adjacent individuals are separated by a blank column. The first and second columns in each F1 individual indicate the paternal and maternal chromosomes, respectively. In a column, a color change from green to blue denotes the occurrence of a recombination event, and gray indicates missing data. Figure S2: Heat maps of the linkage map. Each cell represents the recombination rate of pair-wise markers. Yellow indicates a lower recombination rate, suggesting a stronger linkage between markers. Purple represents the opposite situation.
