# Supplementary figures and images for: Genetic Mapping of Prince Rupprecht’s Larch (Larix principis-rupprechtii Mayr) by Specific-Locus Amplified Fragment Sequencing
Source: Genes (Basel). 2019 Jul 31;10(8):583. doi: 10.3390/genes10080583 (PMC6723236; doi:10.3390/genes10080583)

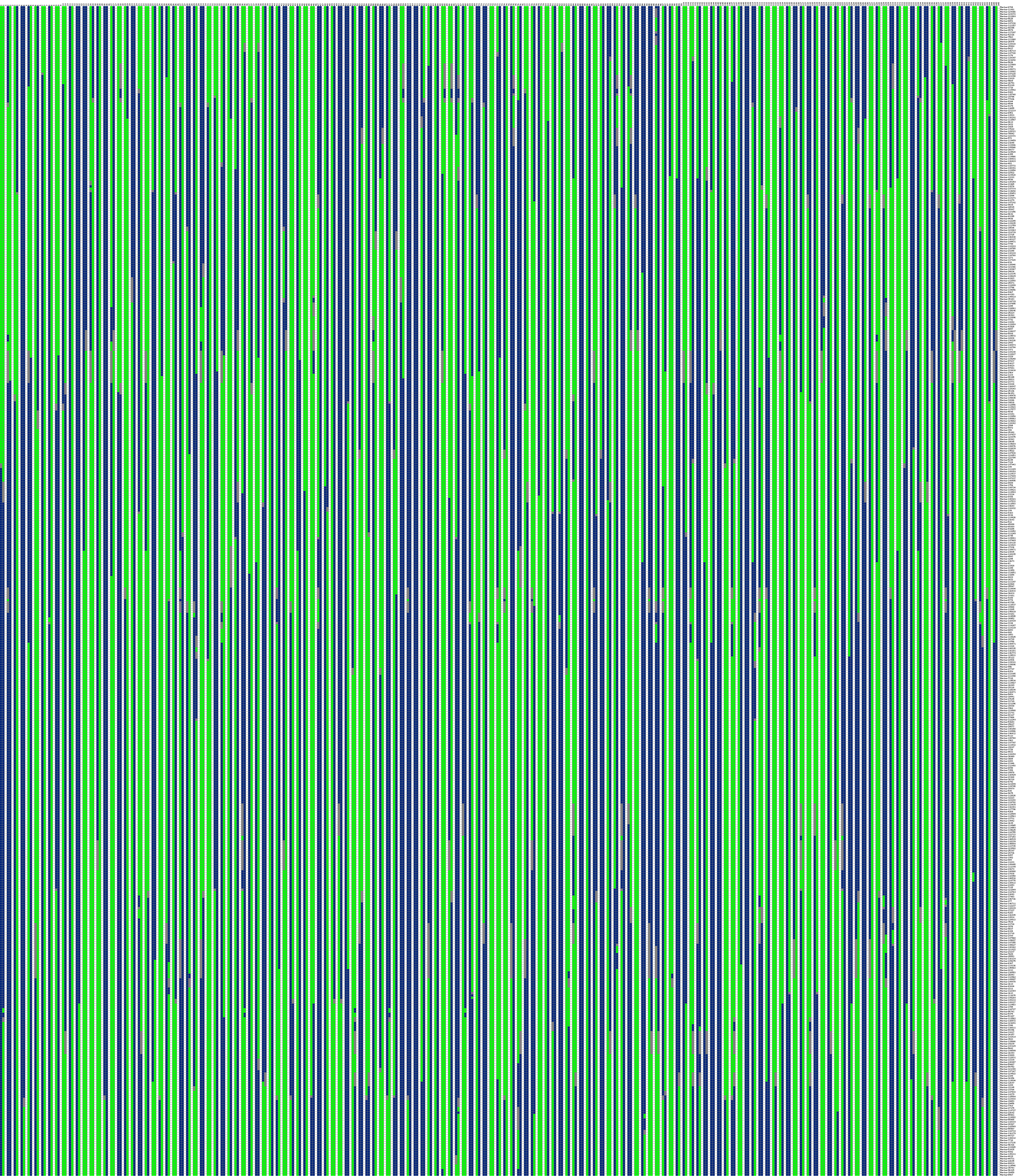

Supplement: Supplementary file 1 [file genes-10-00583-s001.zip › Figure S1/LG1.haploMap.png]

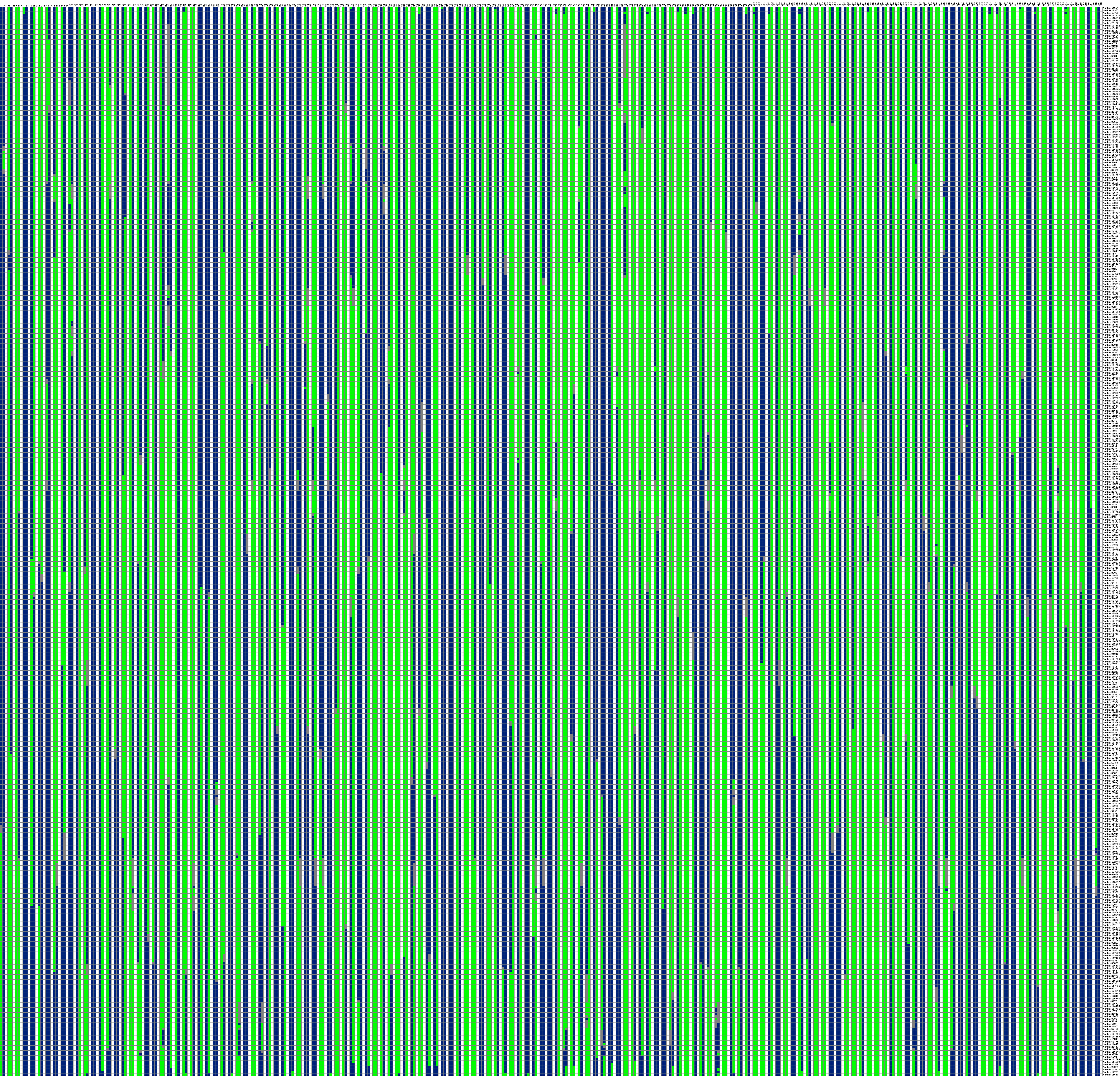

Supplement: Supplementary file 1 [file genes-10-00583-s001.zip › Figure S1/LG10.haploMap.png]

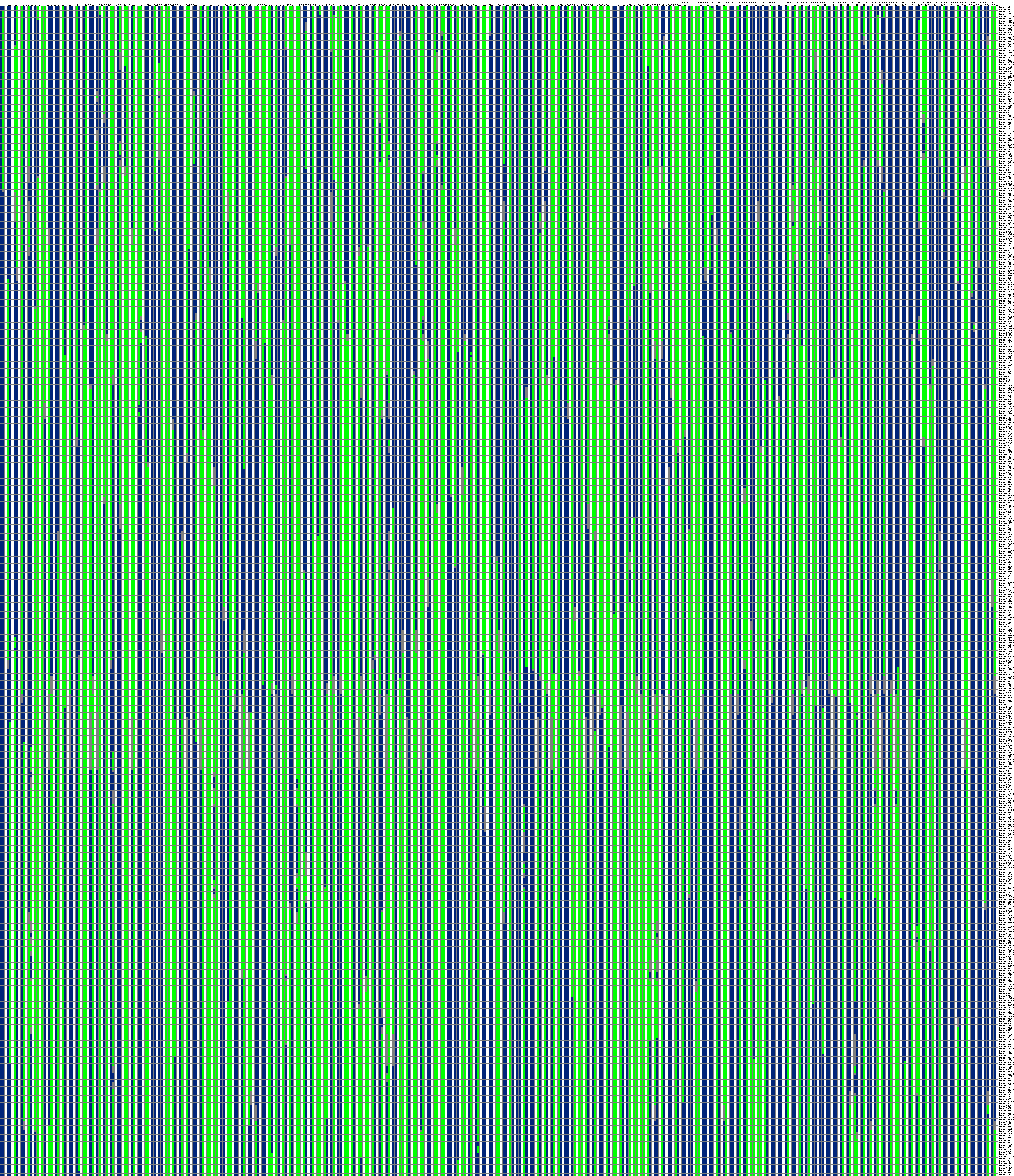

Supplement: Supplementary file 1 [file genes-10-00583-s001.zip › Figure S1/LG11.haploMap.png]

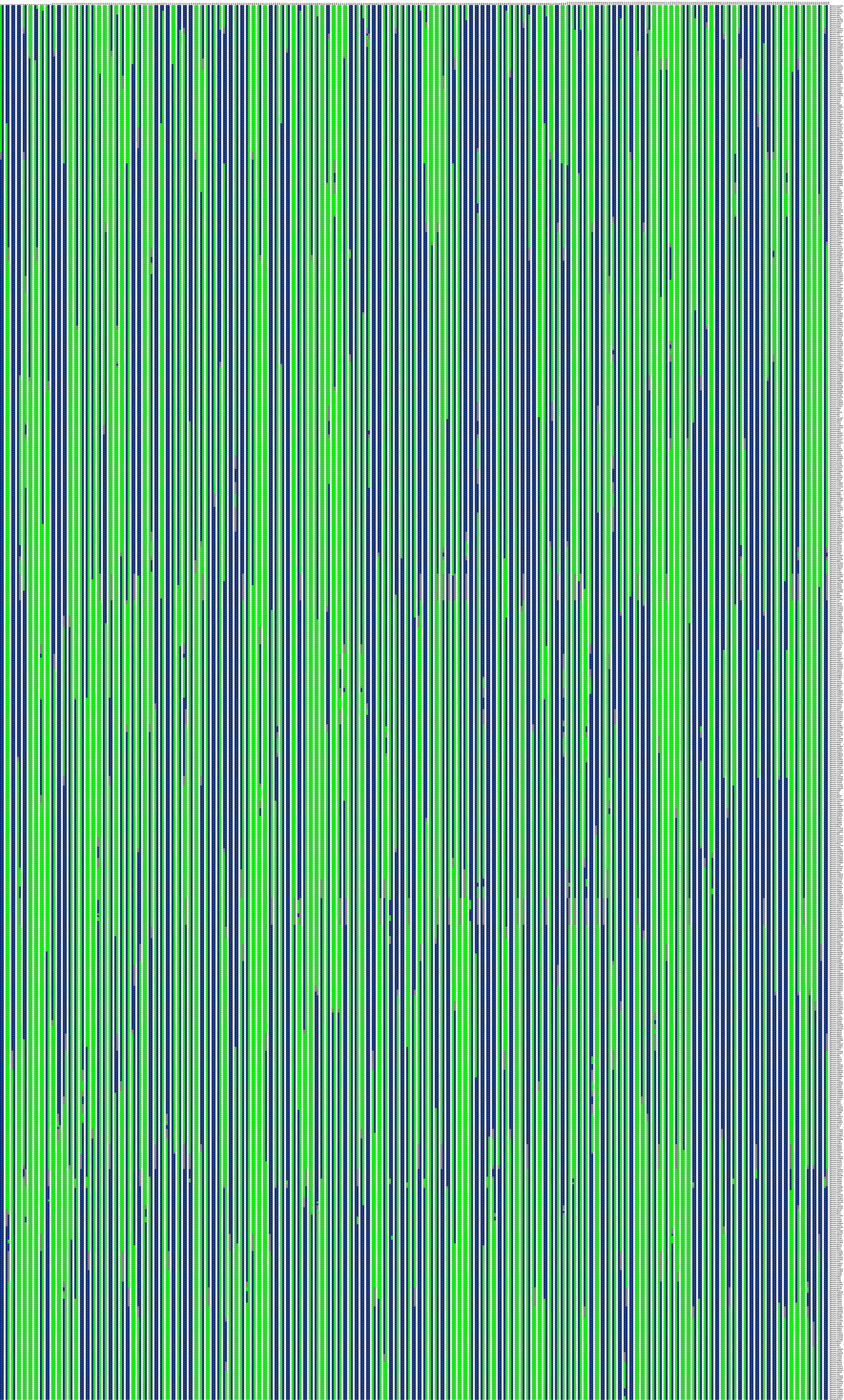

Supplement: Supplementary file 1 [file genes-10-00583-s001.zip › Figure S1/LG12.haploMap.png]

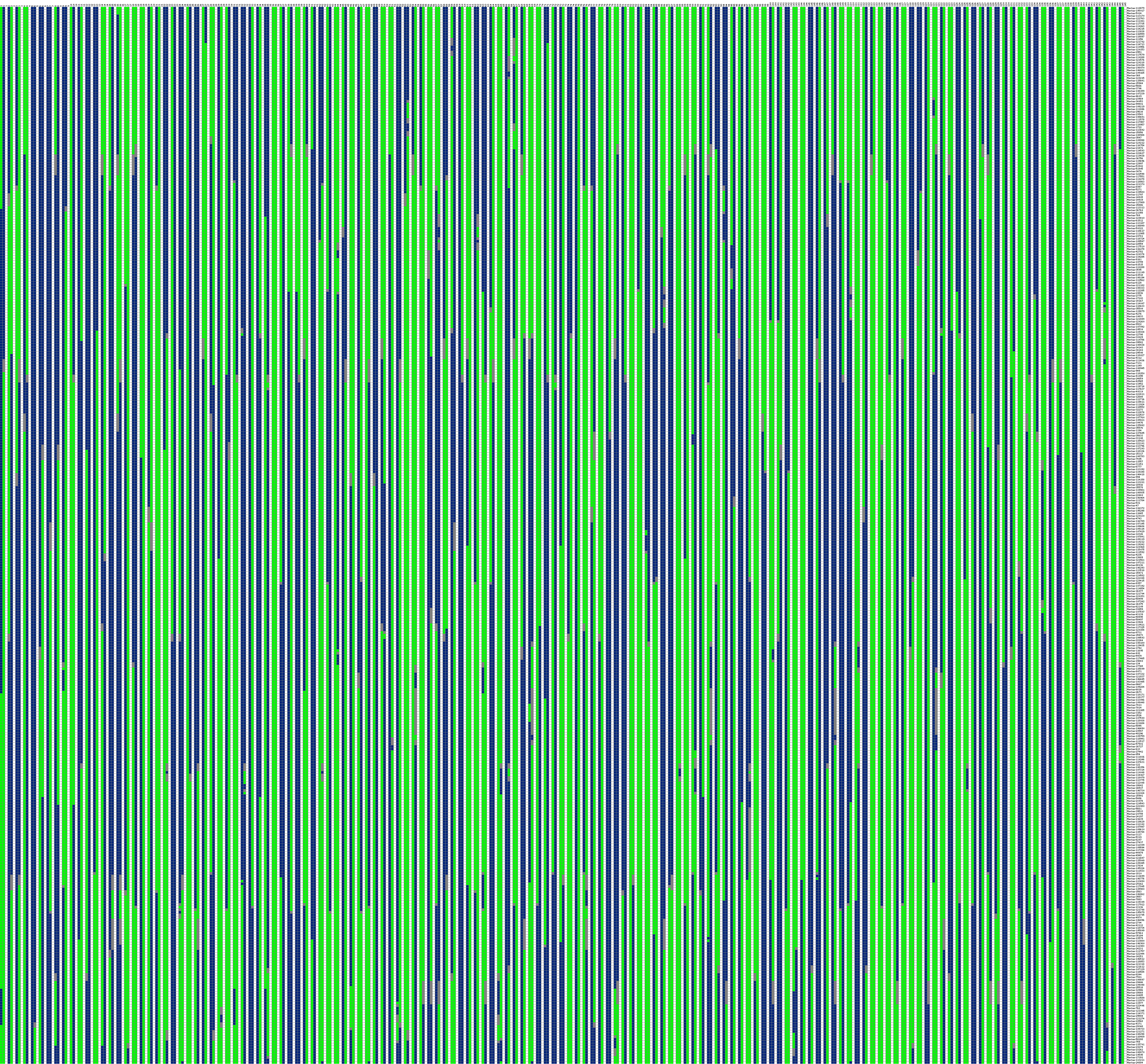

Supplement: Supplementary file 1 [file genes-10-00583-s001.zip › Figure S1/LG2.haploMap.png]

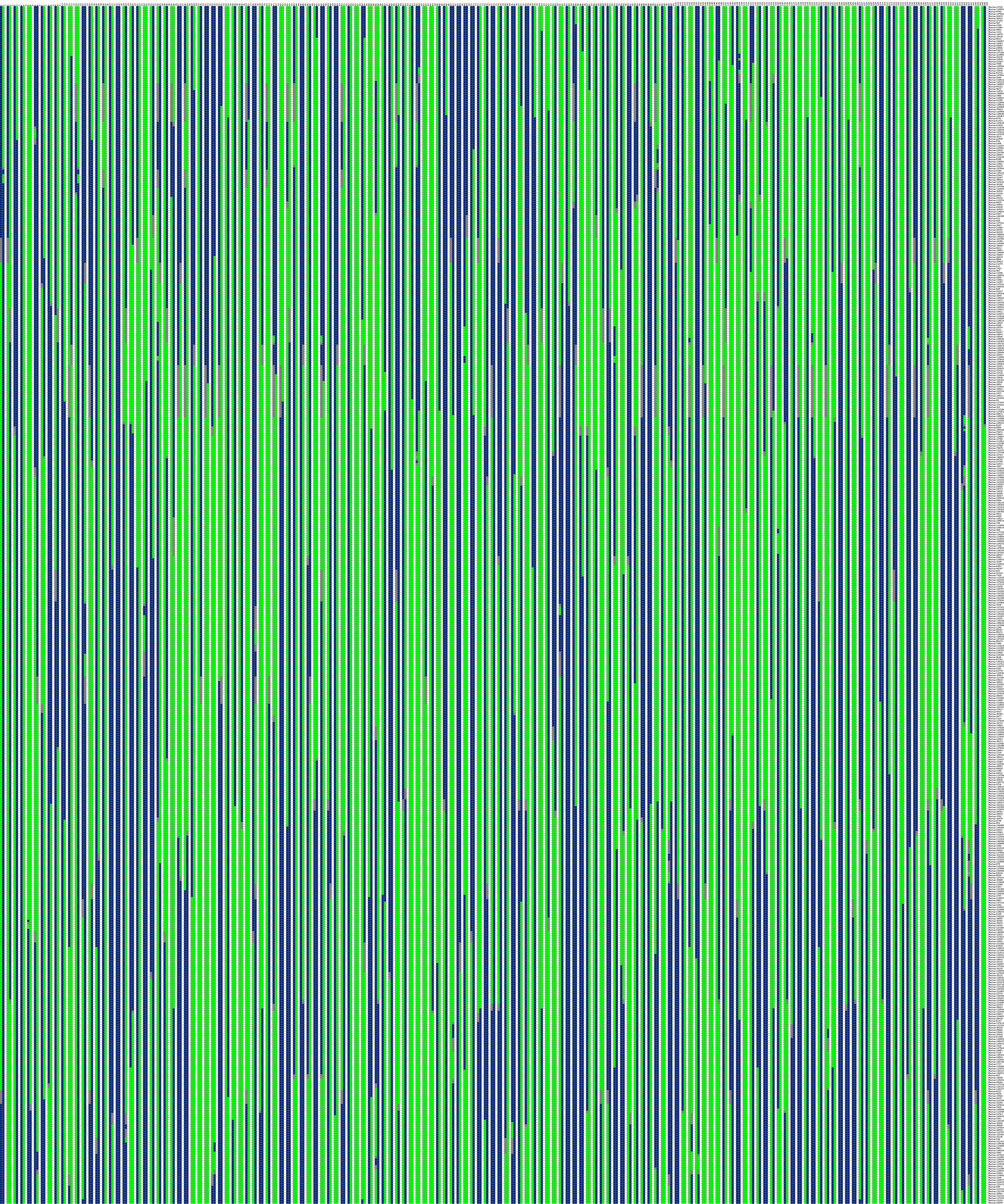

Supplement: Supplementary file 1 [file genes-10-00583-s001.zip › Figure S1/LG3.haploMap.png]

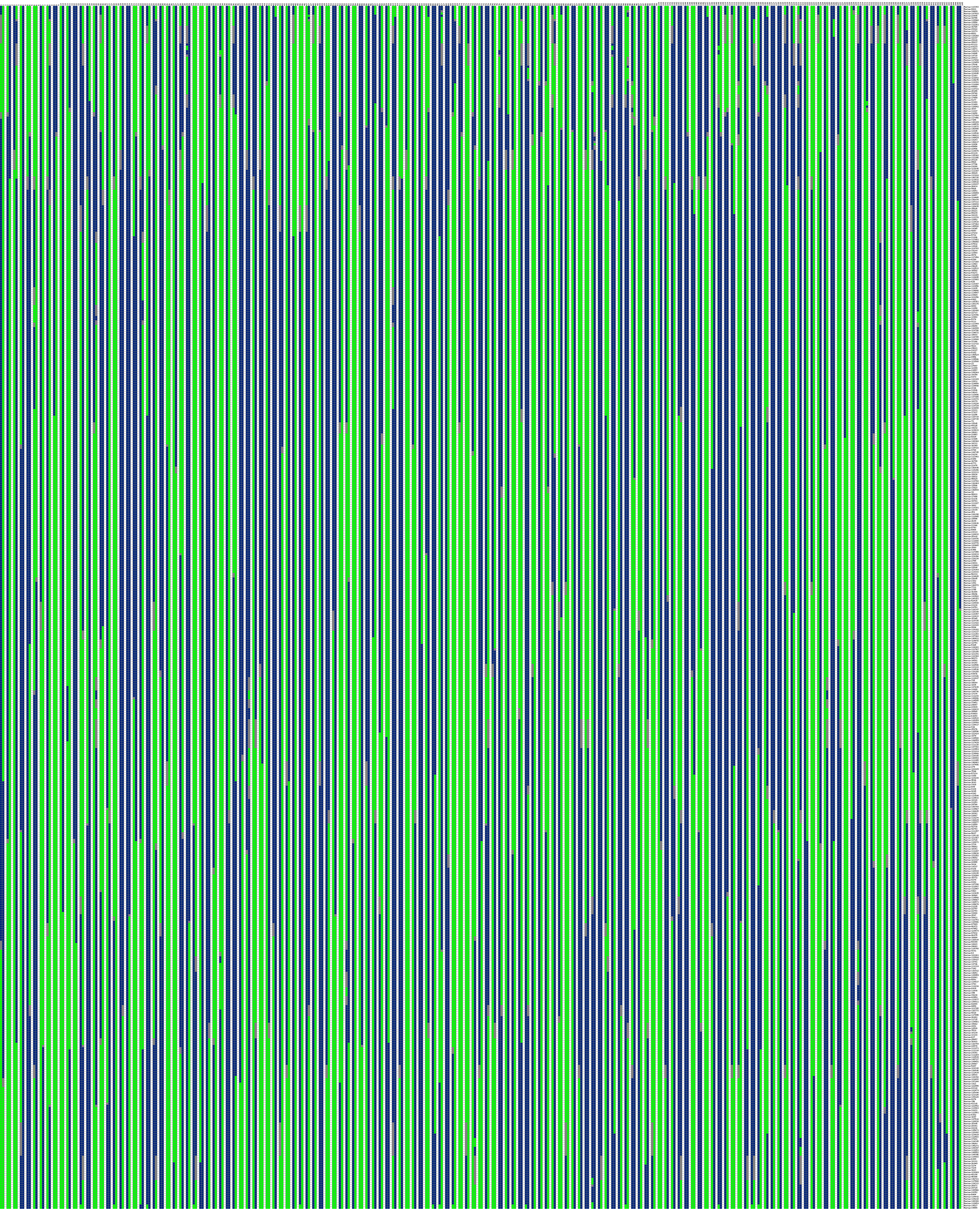

Supplement: Supplementary file 1 [file genes-10-00583-s001.zip › Figure S1/LG4.haploMap.png]

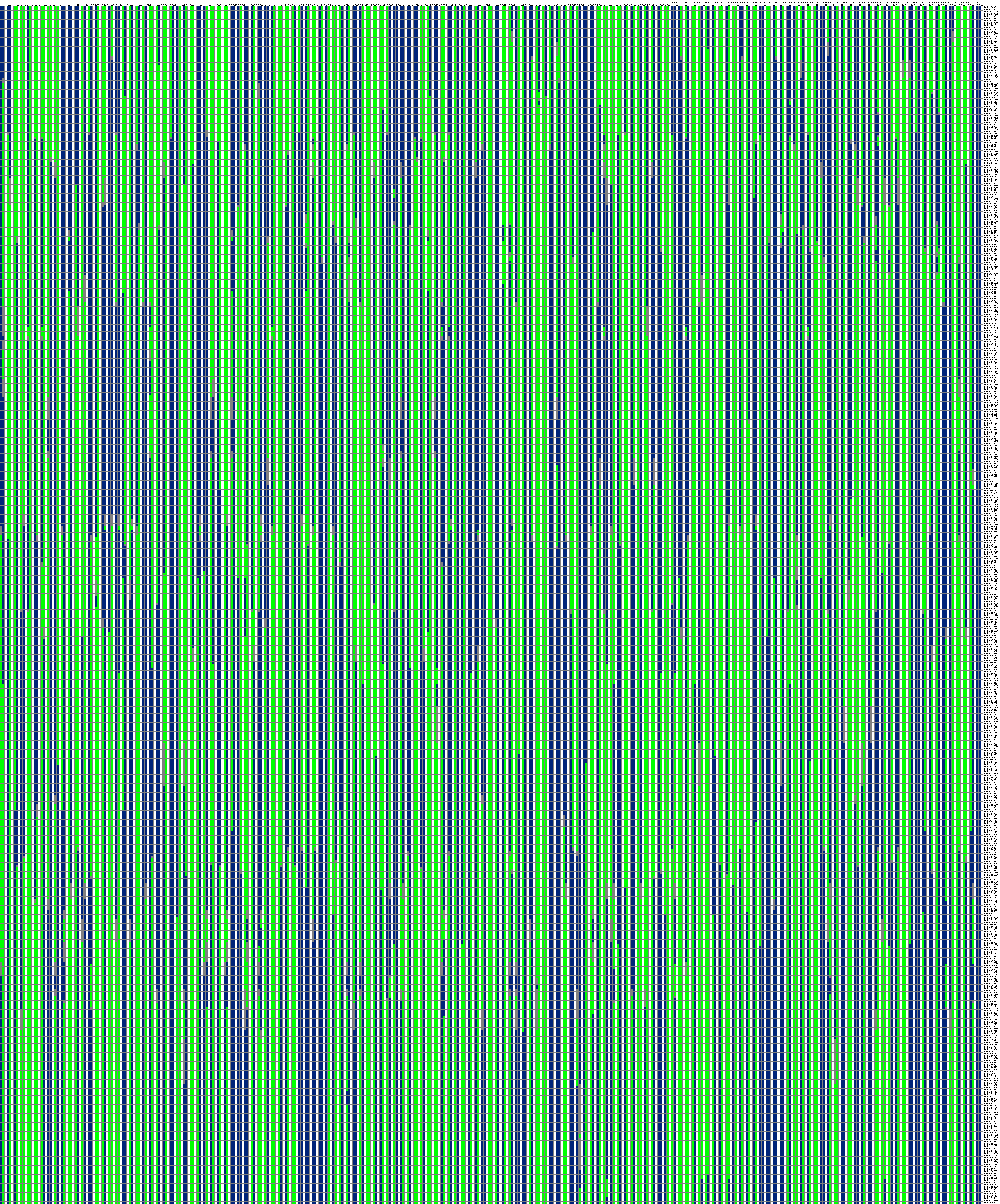

Supplement: Supplementary file 1 [file genes-10-00583-s001.zip › Figure S1/LG5.haploMap.png]

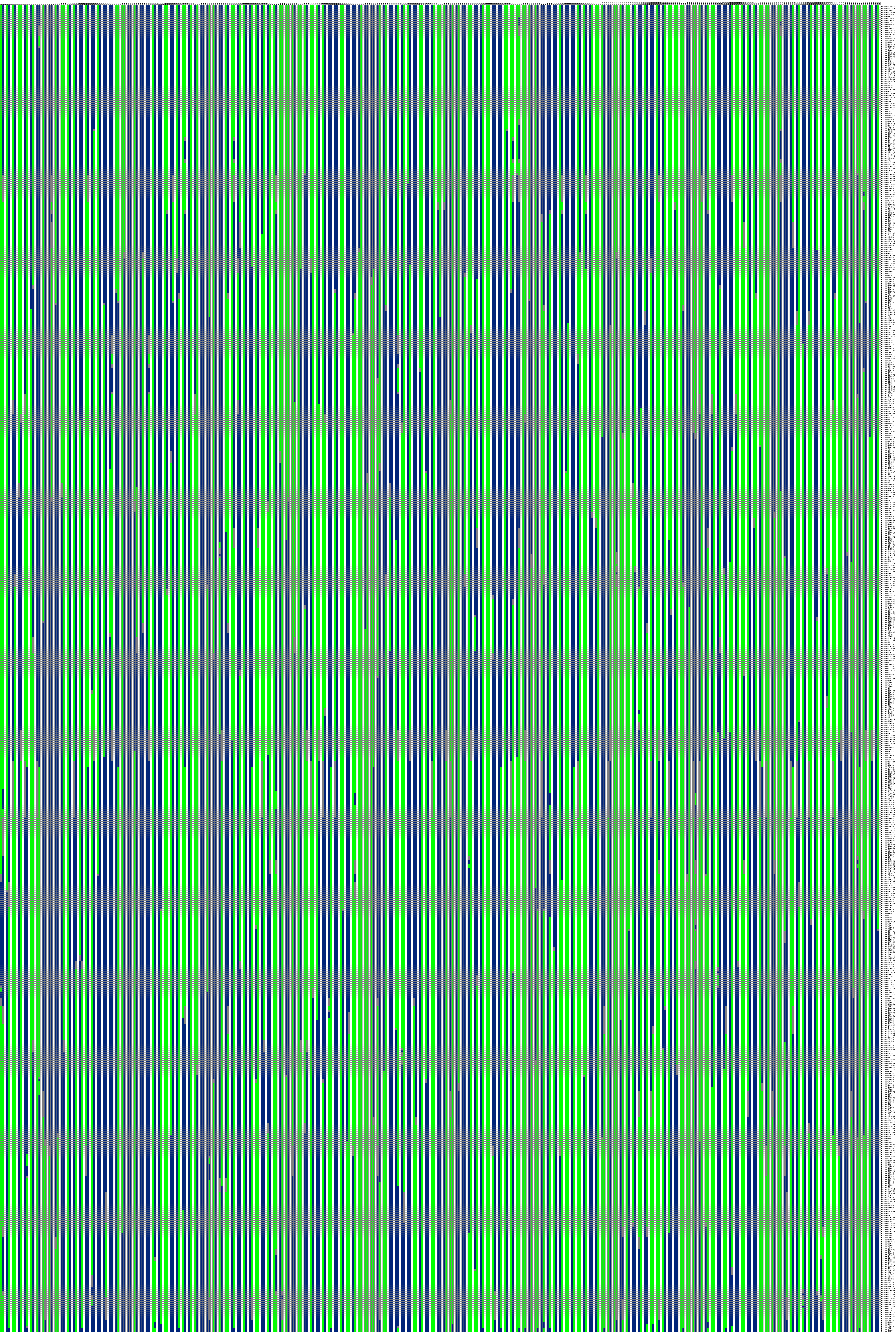

Supplement: Supplementary file 1 [file genes-10-00583-s001.zip › Figure S1/LG6.haploMap.png]

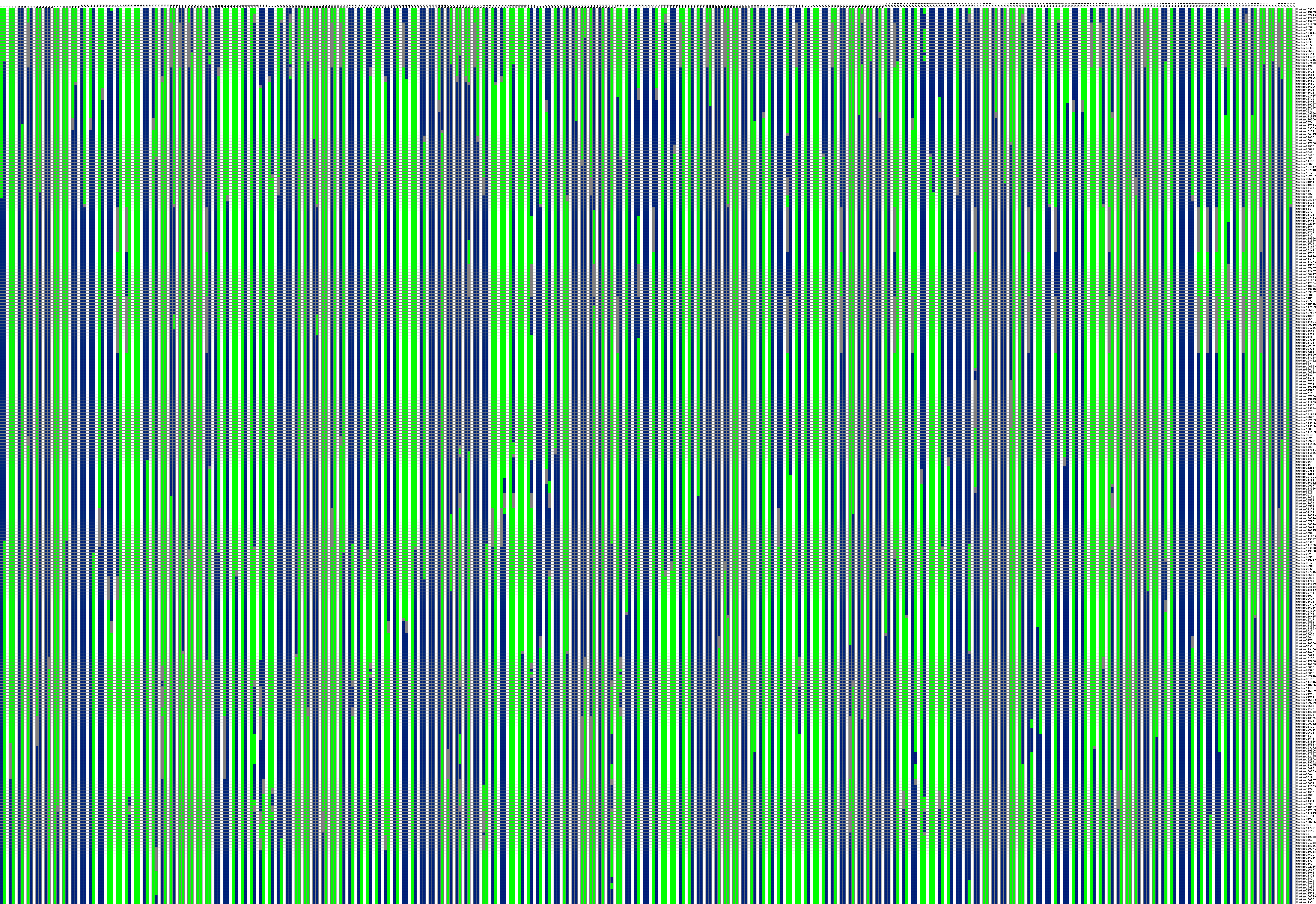

Supplement: Supplementary file 1 [file genes-10-00583-s001.zip › Figure S1/LG7.haploMap.png]

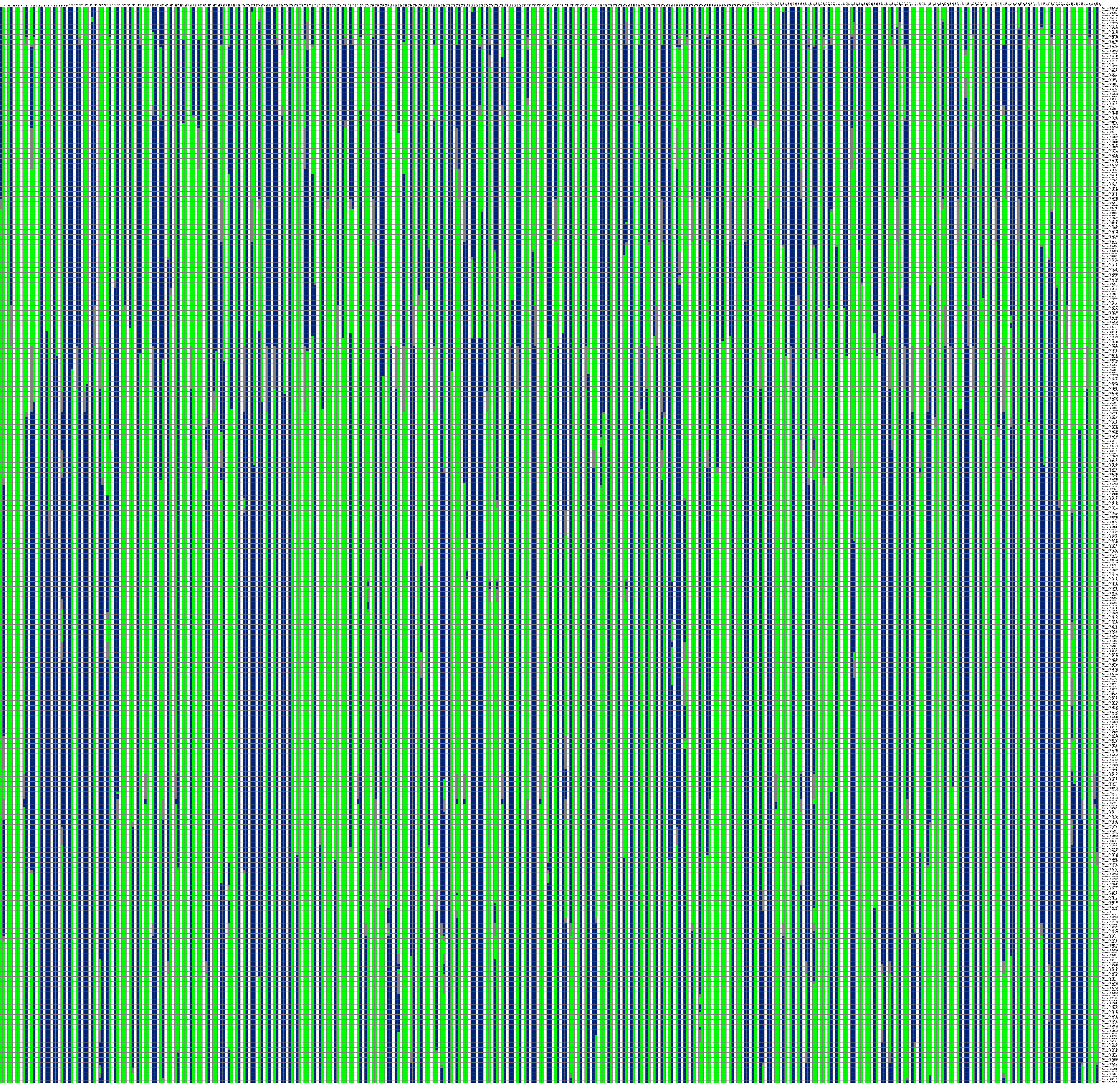

Supplement: Supplementary file 1 [file genes-10-00583-s001.zip › Figure S1/LG8.haploMap.png]

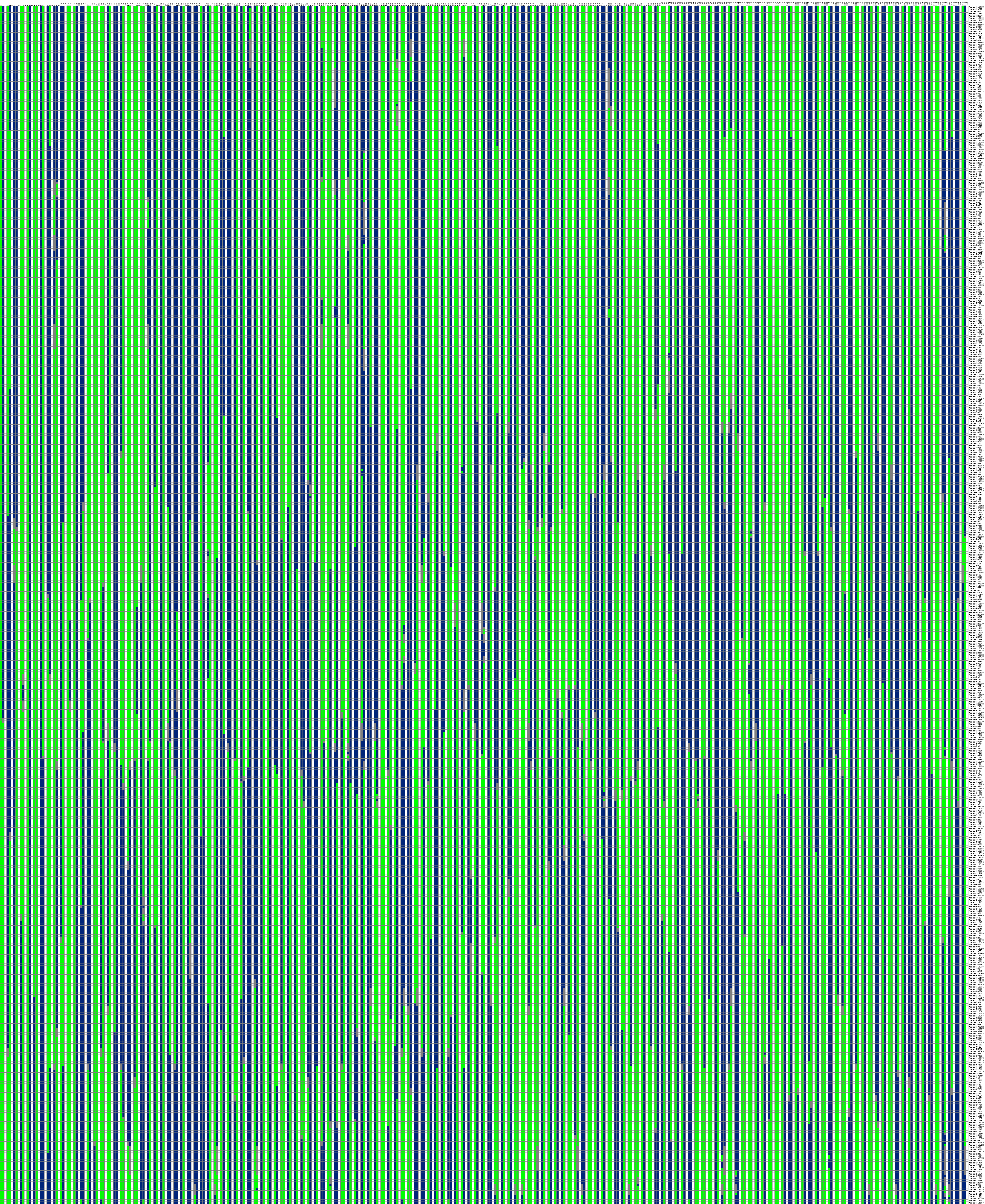

Supplement: Supplementary file 1 [file genes-10-00583-s001.zip › Figure S1/LG9.haploMap.png]

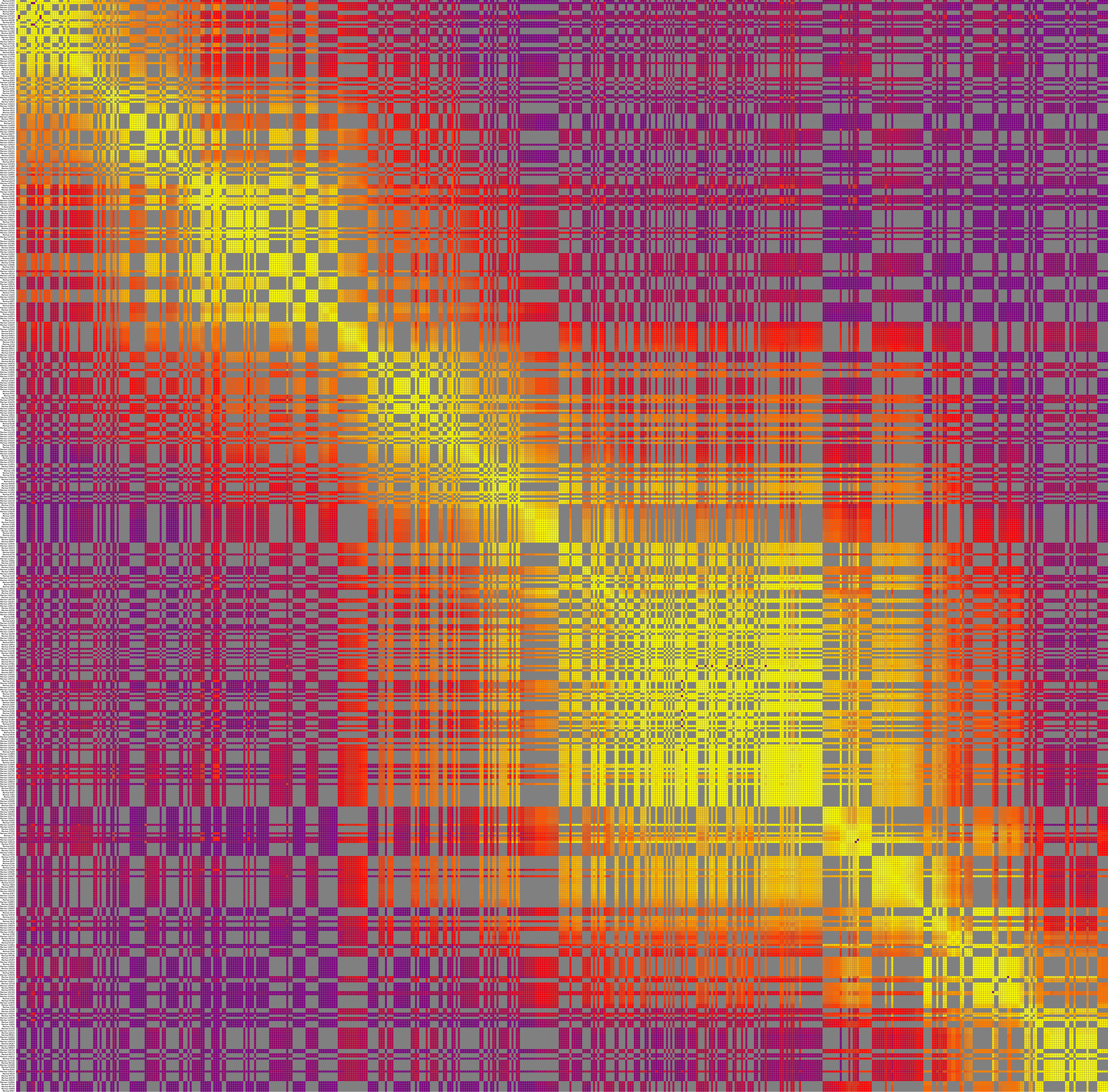

Supplement: Supplementary file 1 [file genes-10-00583-s001.zip › Figure S2/LG1.heatMap.png]

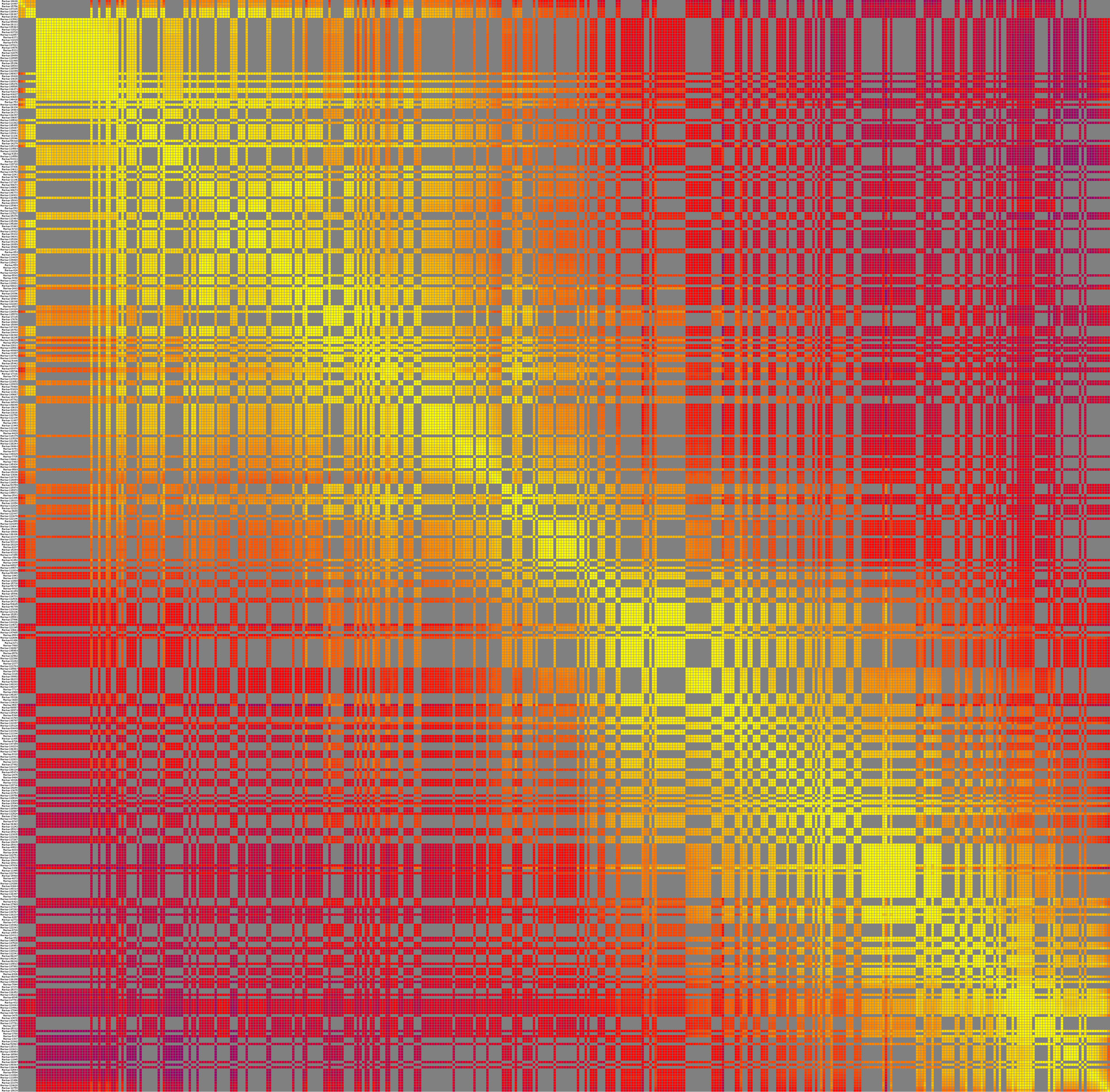

Supplement: Supplementary file 1 [file genes-10-00583-s001.zip › Figure S2/LG10.heatMap.png]

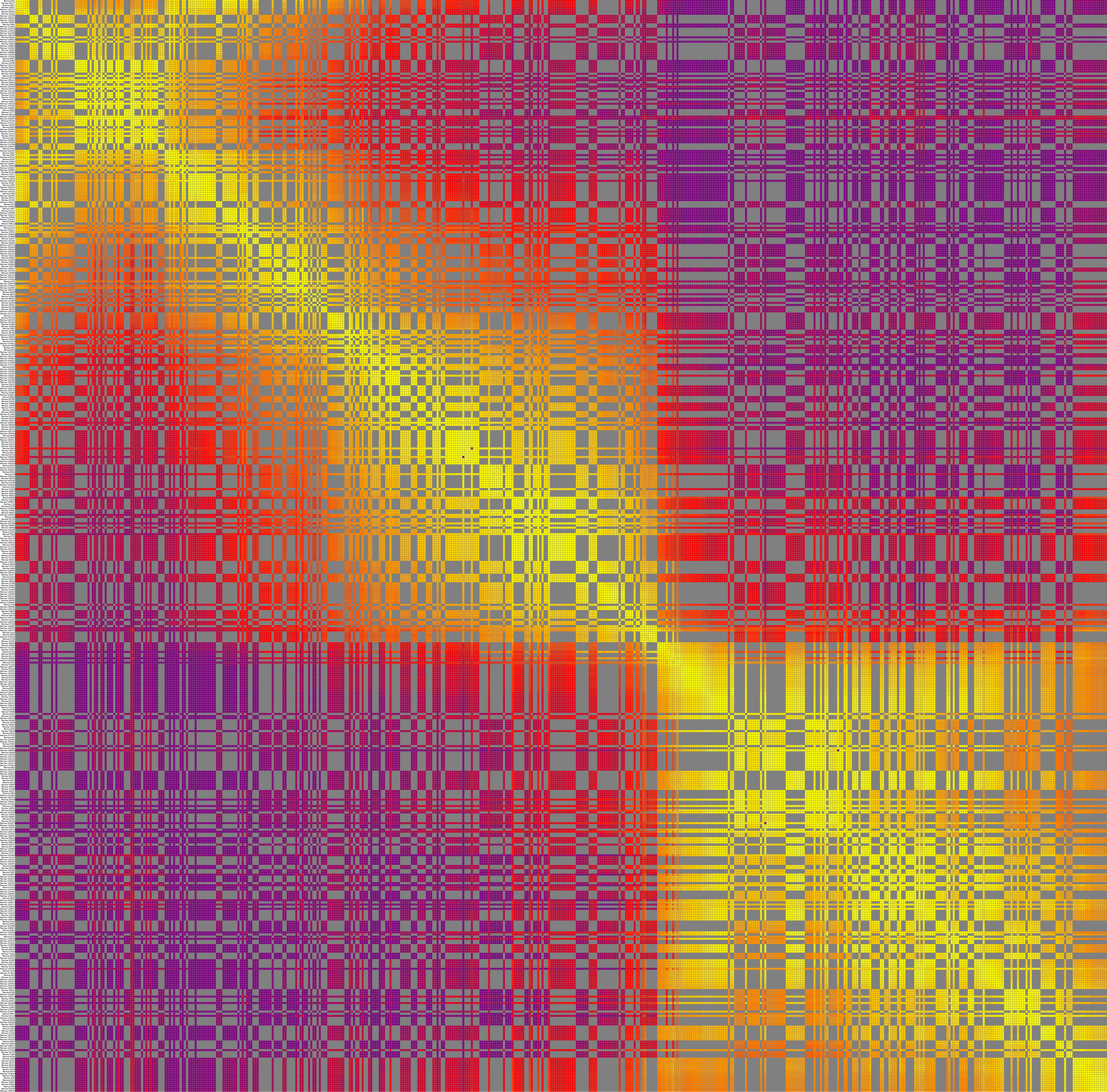

Supplement: Supplementary file 1 [file genes-10-00583-s001.zip › Figure S2/LG11.heatMap.png]

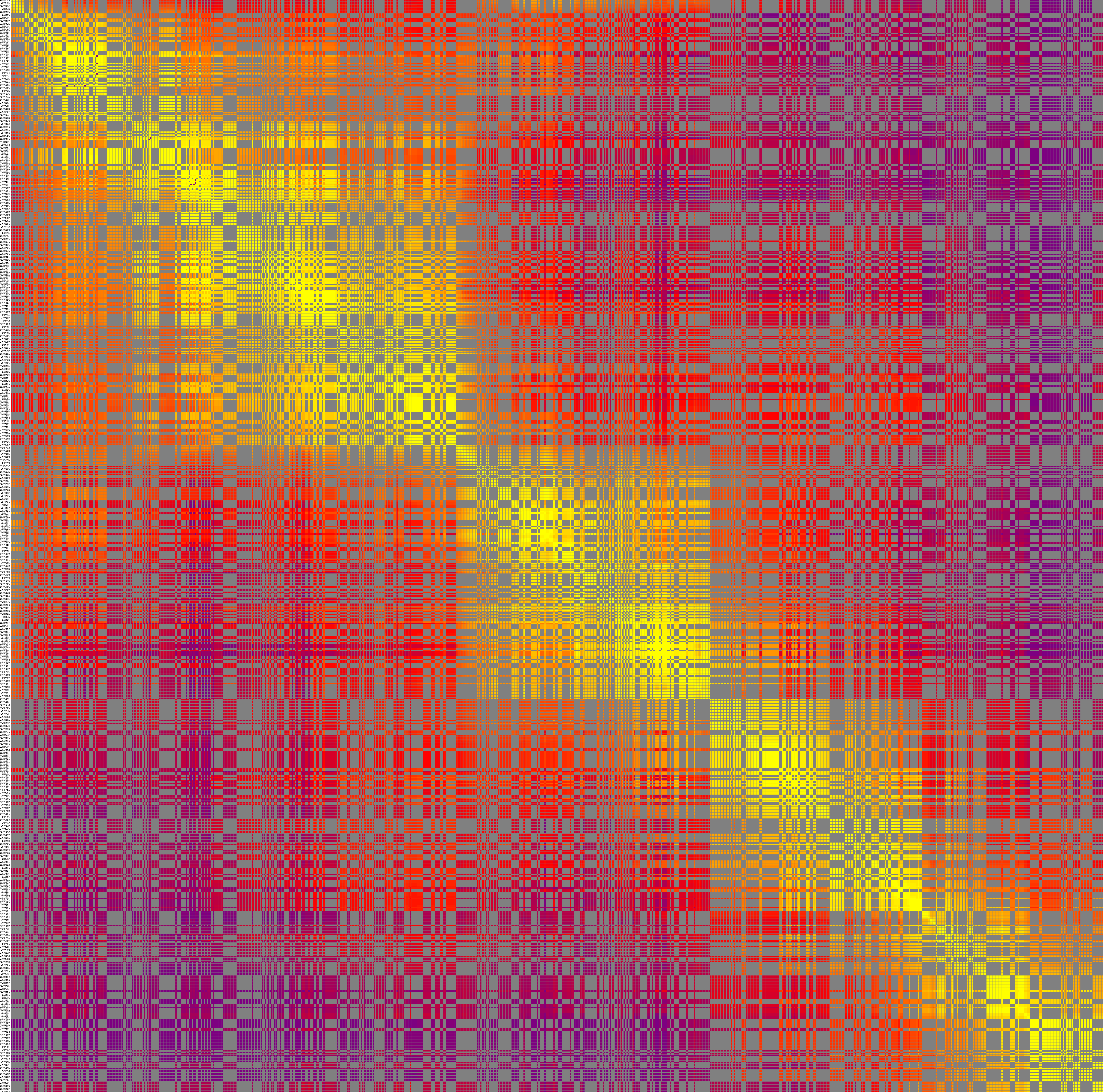

Supplement: Supplementary file 1 [file genes-10-00583-s001.zip › Figure S2/LG12.heatMap.png]

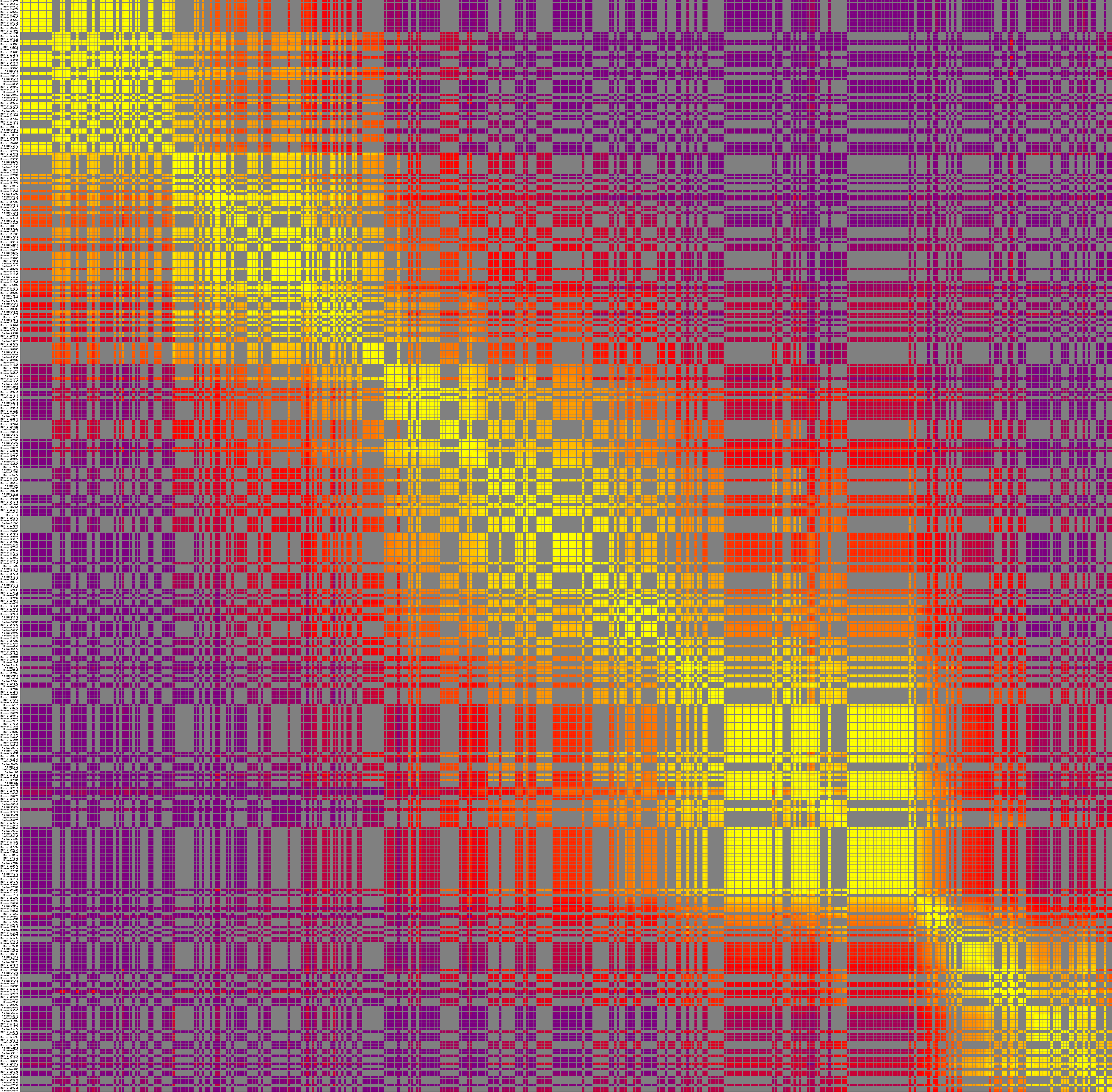

Supplement: Supplementary file 1 [file genes-10-00583-s001.zip › Figure S2/LG2.heatMap.png]

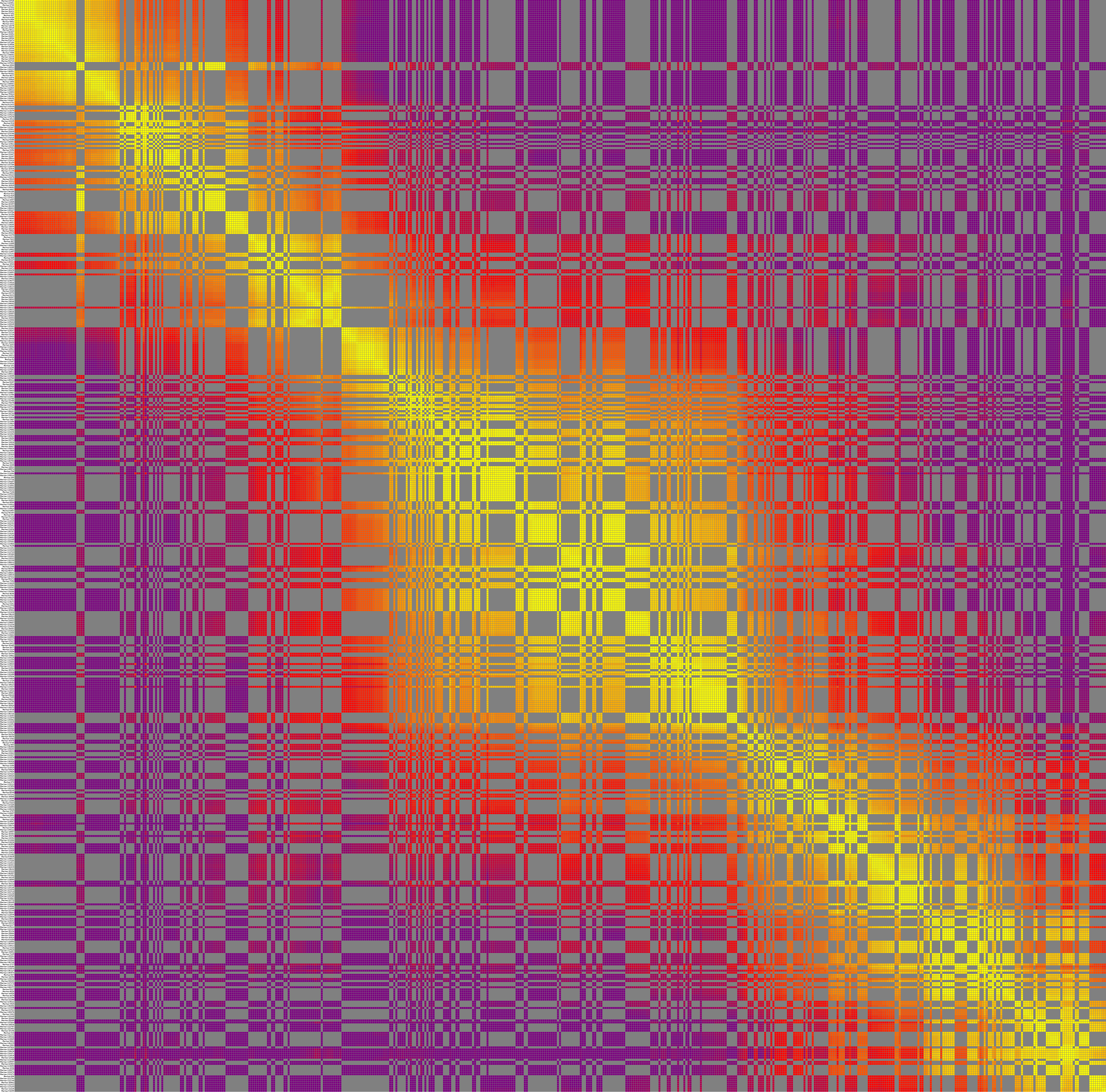

Supplement: Supplementary file 1 [file genes-10-00583-s001.zip › Figure S2/LG3.heatMap.png]

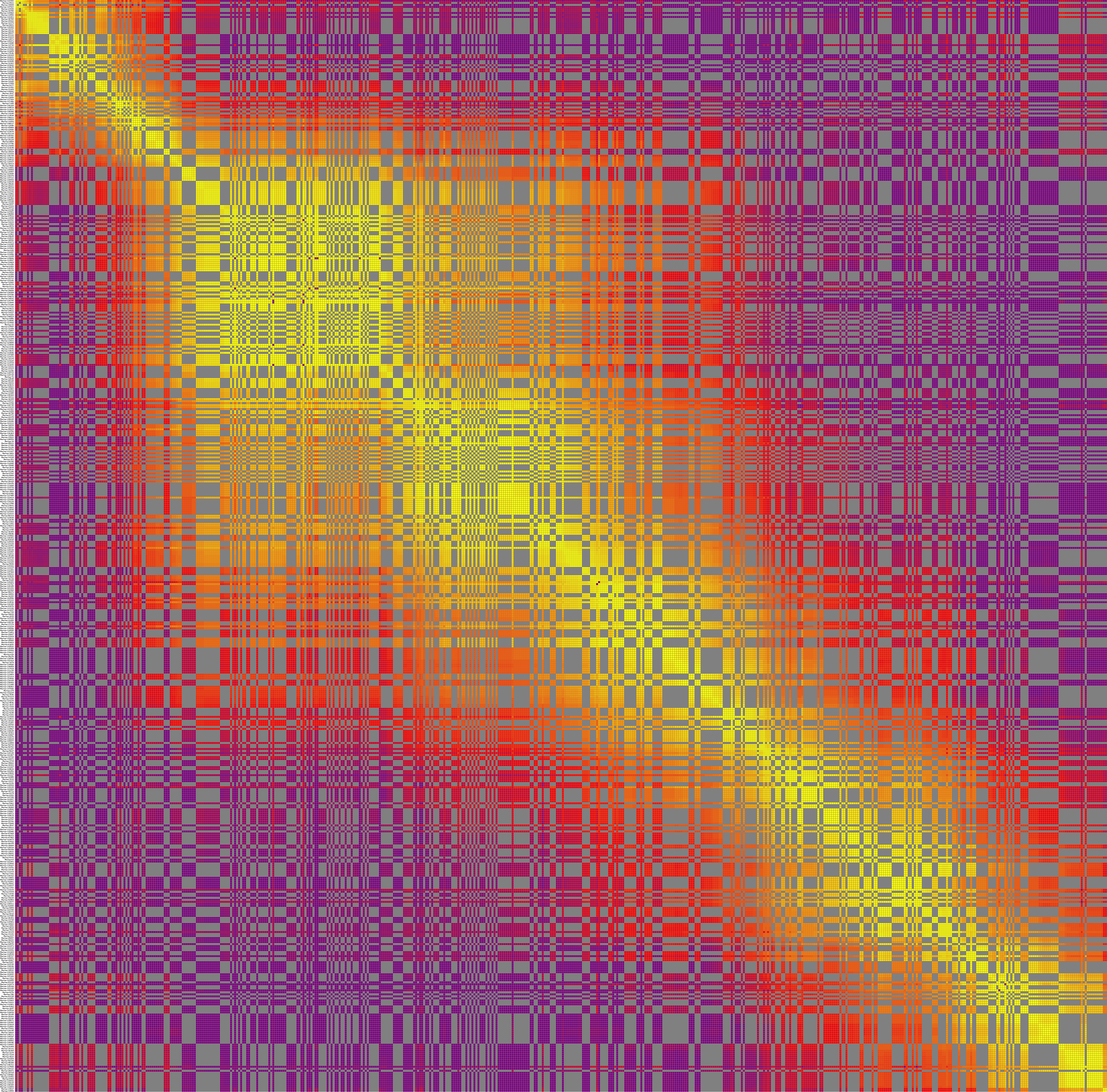

Supplement: Supplementary file 1 [file genes-10-00583-s001.zip › Figure S2/LG4.heatMap.png]

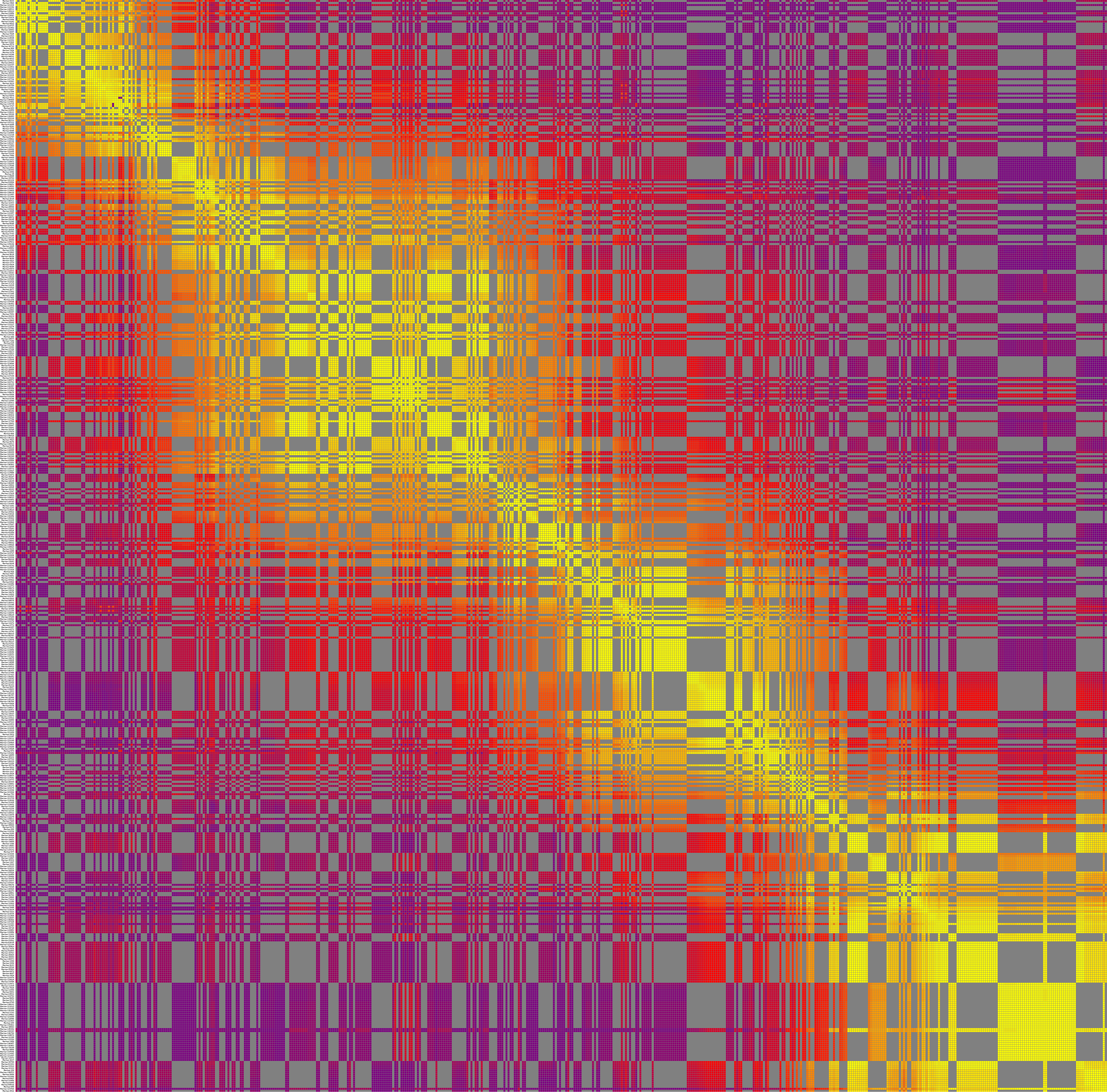

Supplement: Supplementary file 1 [file genes-10-00583-s001.zip › Figure S2/LG5.heatMap.png]

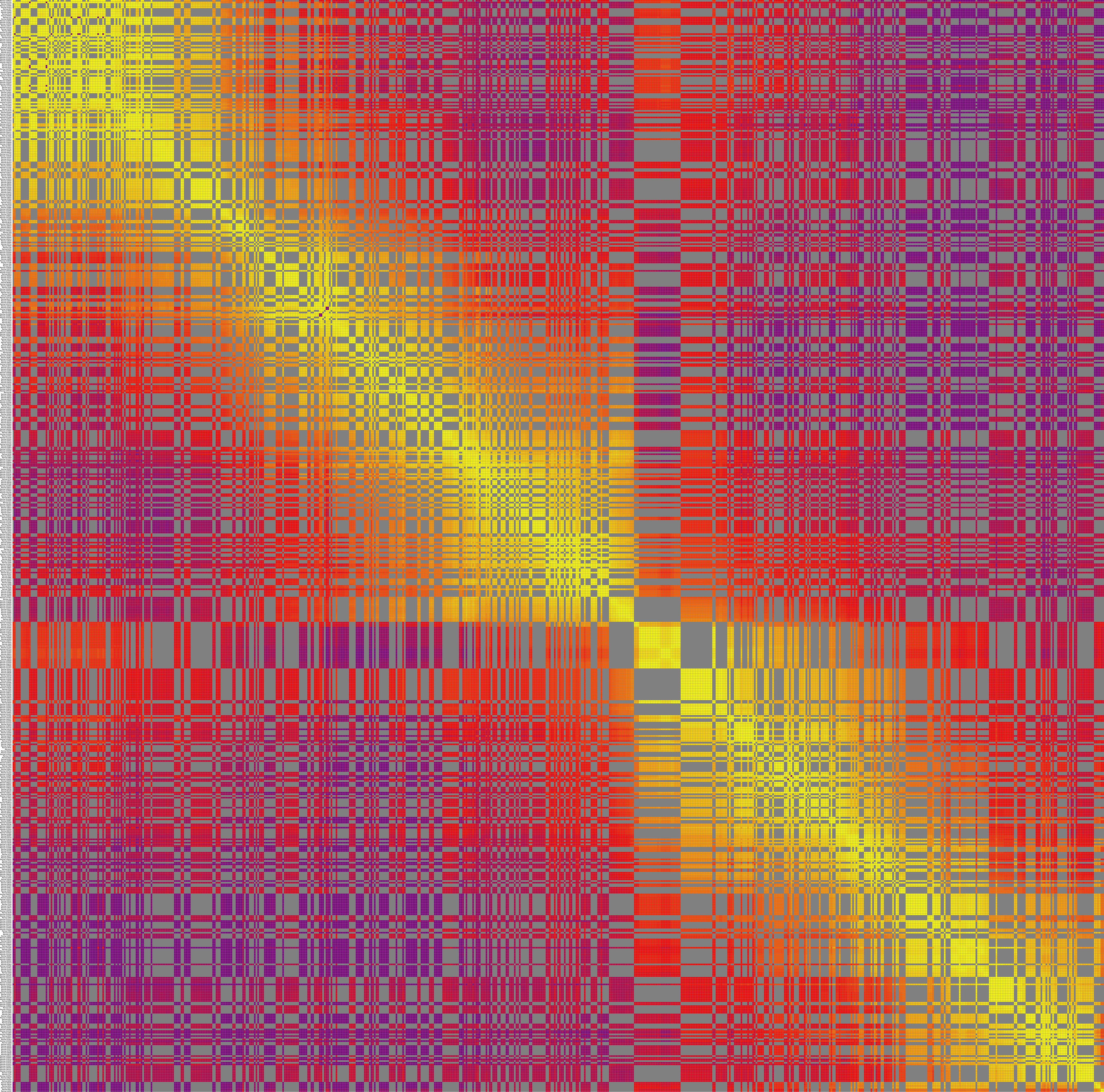

Supplement: Supplementary file 1 [file genes-10-00583-s001.zip › Figure S2/LG6.heatMap.png]

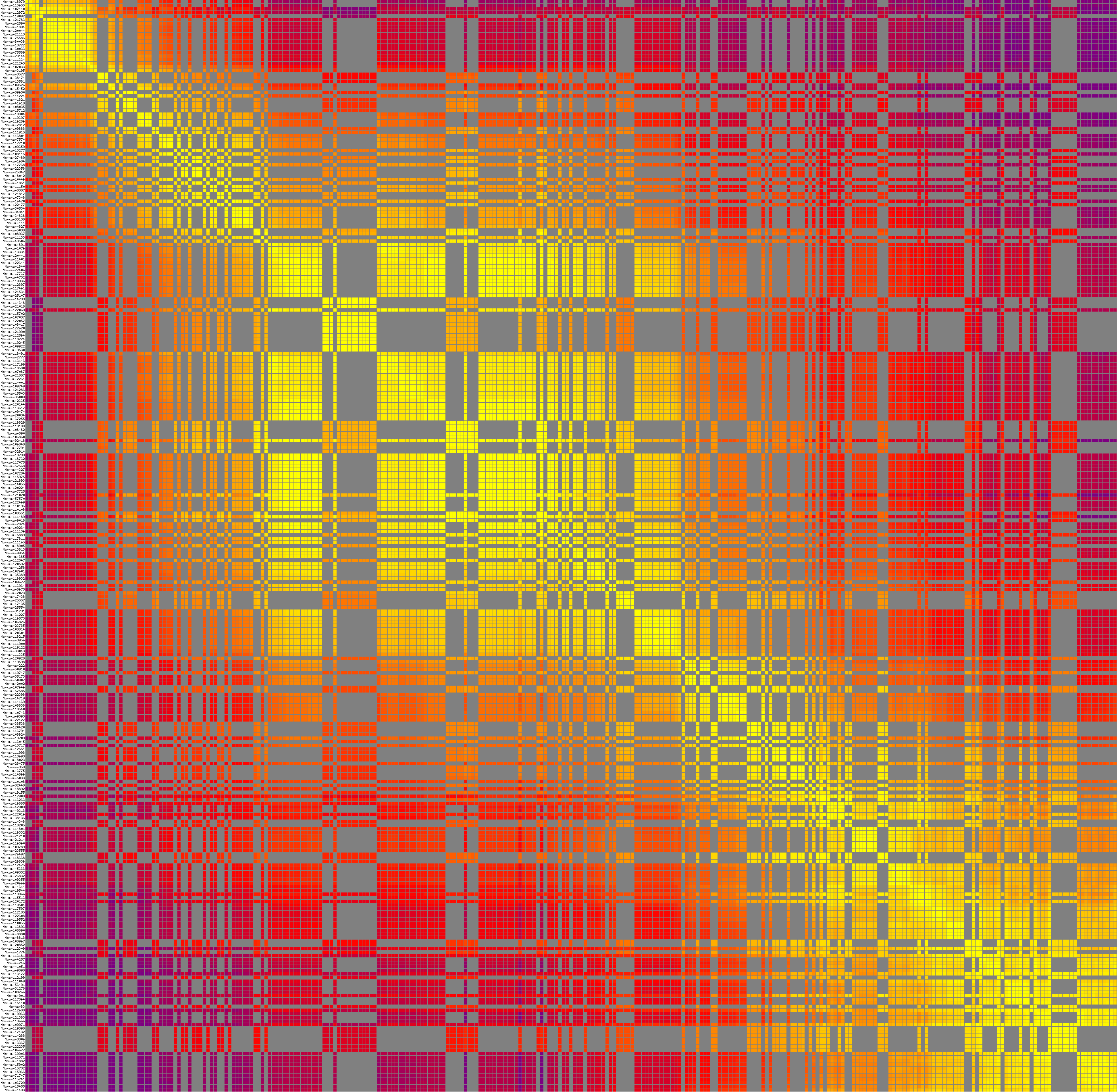

Supplement: Supplementary file 1 [file genes-10-00583-s001.zip › Figure S2/LG7.heatMap.png]

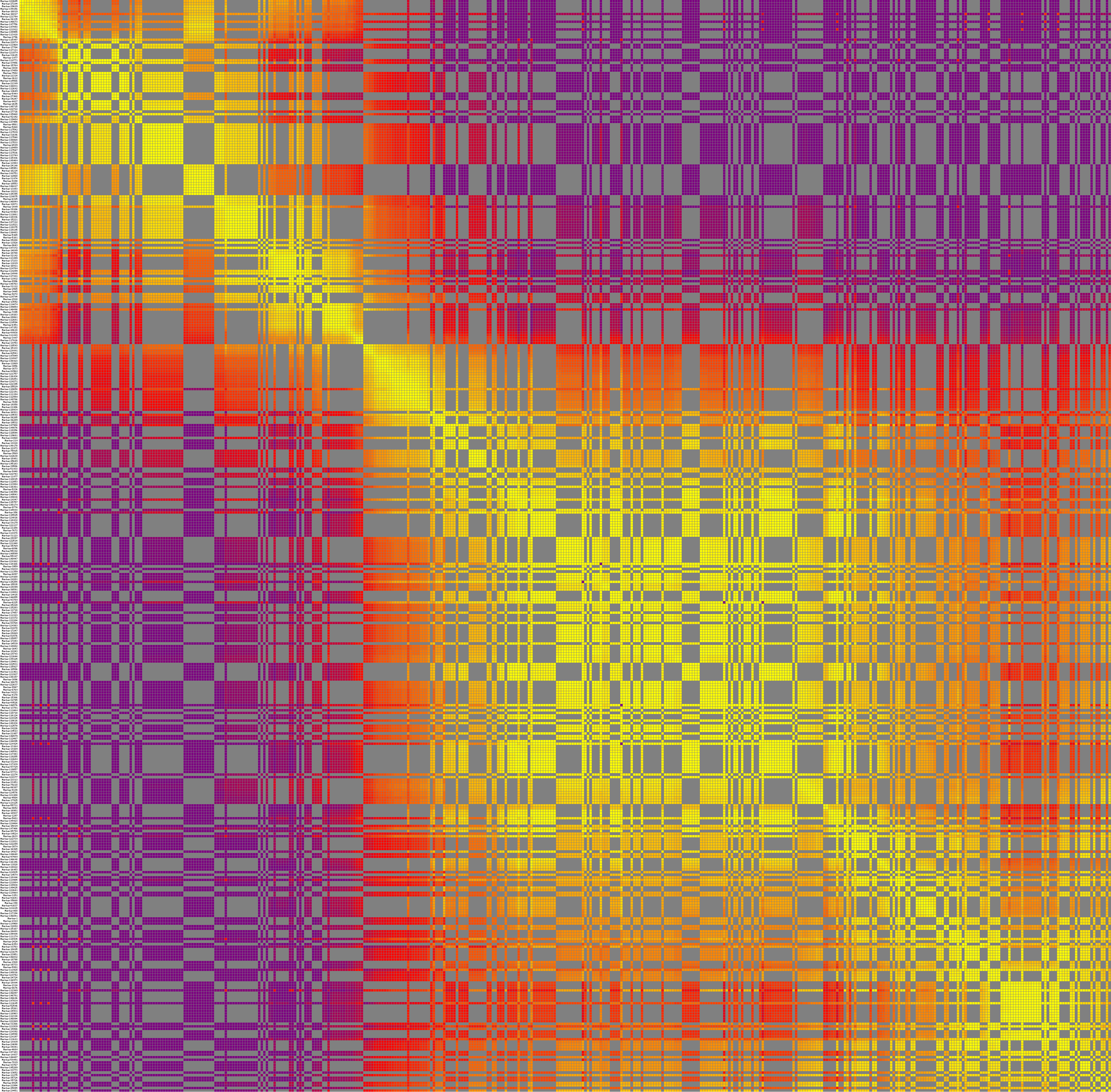

Supplement: Supplementary file 1 [file genes-10-00583-s001.zip › Figure S2/LG8.heatMap.png]

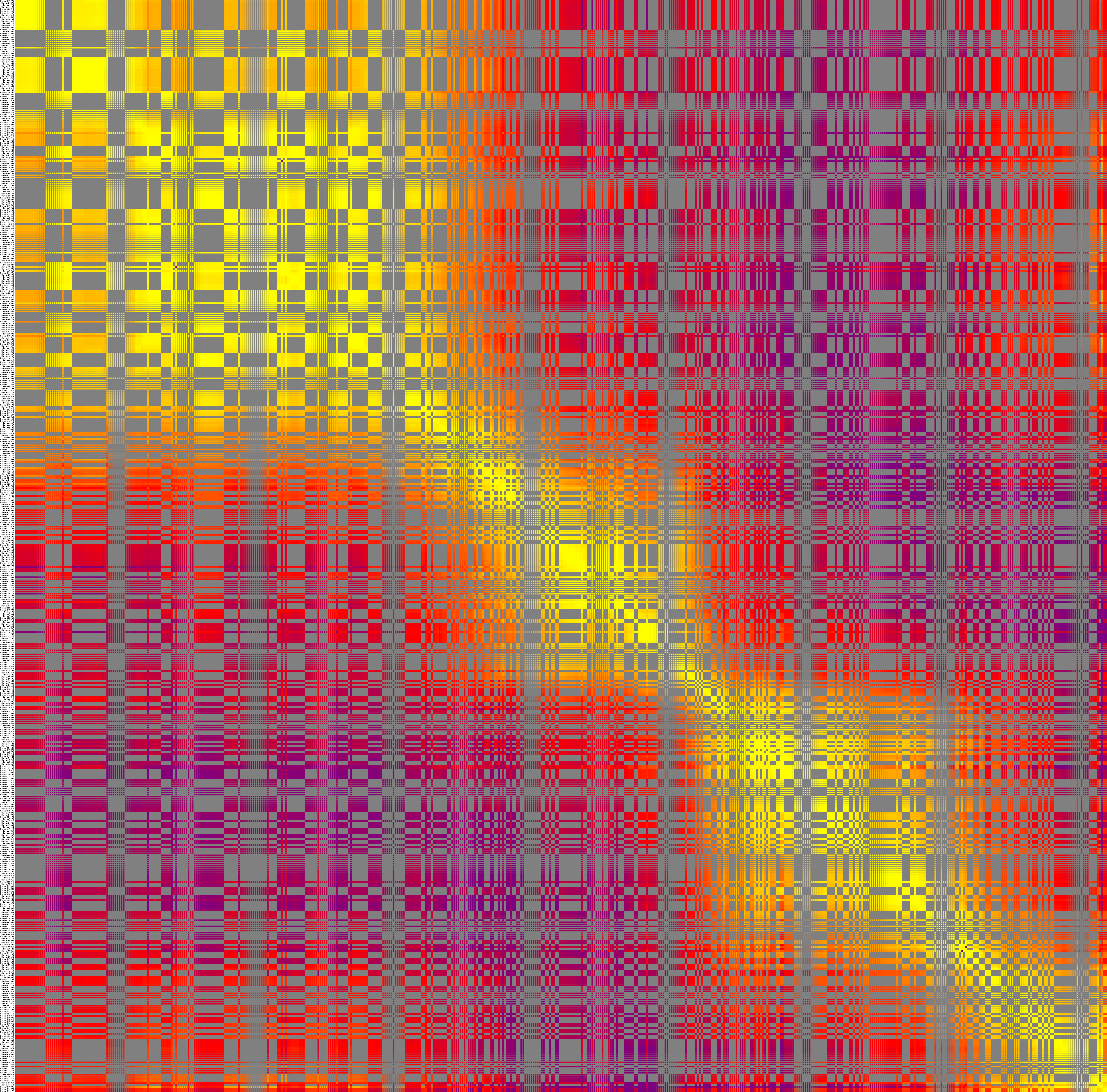

Supplement: Supplementary file 1 [file genes-10-00583-s001.zip › Figure S2/LG9.heatMap.png]
